# Effects of LED spectral compositions on yield, growth, and nutritional quality of basil microgreens in indoor vertical farming

**DOI:** 10.1371/journal.pone.0352317

**Published:** 2026-07-07

**Authors:** Sibel Balik, Abdullah Aldiyab, Tuğçe Temtek, Boran İkiz, Hayriye Yildiz Dasgan, Nazim S. Gruda

**Affiliations:** 1 Department of Horticulture, Faculty of Agriculture, University of Cukurova, Adana, Turkey; 2 Institute of Plant Sciences and Resource Conservation, Division of Horticultural Sciences, University of Bonn, Bonn, Germany; Institute for Biological Research, University of Belgrade, SERBIA

## Abstract

Light quality, particularly the spectral composition emitted by LEDs, modulate growth dynamics, physiological processes, and the nutritional attributes of microgreens cultivated under controlled environments. In this study, the effects of three distinct LED spectra on growth performance, pigmentation, and nutritional composition of basil (*Ocimum basilicum* L.) microgreens were assessed within an indoor vertical farming system. The lighting treatments included: (LED1) 70% red (660 nm) + 30% blue (450 nm), (LED 2) a full-spectrum PAR range (400–700 nm), and (LED 3) 65% red (660 nm), 25% blue (450 nm), 5% white (broad spectrum around 400–700 nm), and 5% far-red (730 nm). Key parameters assessed were plant height, hypocotyl length, stem diameter, individual plant fresh weight, yield, color values (L* = luminosity, a* = red-green axis, b* = blue-yellow axis), dry matter content, macro and micronutrient levels, pH (hydrogen ion concentration) and titratable acidity (total acidity), soluble solid content (SSC), electrical conductivity (EC), and concentrations of oil, total phenols, flavonoids, and vitamin C. Among the treatments, LED 3 significantly enhanced plant height, hypocotyl length, yield, total phenol content, acidity, EC, and color brightness (L*), highlighting its superior overall performance. LED 2 was most effective in increasing vitamin C, flavonoid, and oil content, while LED 1 promoted higher dry matter and mineral contents. These findings emphasize the importance of optimizing LED spectra to improve both productivity and nutritional quality. Based on its consistent advantages across multiple parameters, LED 3 is the most advantageous in terms of yield, biomass accumulation, and visual quality, highlighting its potential suitability for commercial microgreen cultivation in controlled environment systems. Further research is needed to validate these findings across different cultivar-specific responses and environmental conditions.

## 1. Introduction

Microgreens are young seedlings of vegetables and herbs, typically harvested one to two weeks after germination, when the first true leaves emerge. These tender greens are considered a functional food due to their dense concentration of bioactive compounds [1–3]. Compared to their mature counterparts, microgreens may contain 4–40 times higher levels of essential nutrients such as vitamins, antioxidants, minerals, and phytonutrients [4,5]. Their exceptional nutritional profile varies by plant species, with each type offering distinct health-promoting properties and compositional characteristics [3]. As awareness of healthy eating and sustainable agriculture grows, consumer demand for microgreens has significantly increased in recent years, particularly in urban areas and health-conscious markets.

Basil (*Ocimum basilicum L.*) is a widely appreciated medicinal and culinary herb, valued for its characteristic aroma, flavor, and richness in bioactive compounds [6]. It is among the most recognized medicinal herbs, valued for its health-promoting properties, including antioxidant, antimicrobial, anti-inflammatory, anti-cancer, hepatoprotective, anti-stress, and immune-enhancing effects, which are primarily attributed to its rich content of bioactive compounds [7]. Among these, phenolic and flavonoid compounds significantly contribute to its antioxidant properties and overall nutraceutical potential [8,9]. When cultivated as microgreens, basil exhibits an even more concentrated phytochemical profile compared to its mature form, offering enhanced nutritional and functional value [10]. The microgreen stage is associated with elevated levels of health-promoting compounds, making basil microgreens a promising component in the development of nutrient-dense and functional foods [1,11]. Basil microgreens, like many other species, can be successfully grown in controlled indoor environments using LED (light-emitting diode) systems that enable precise manipulation of light quality to enhance growth and nutritional value [1]. Their compact growth form and low spatial demands make them particularly well-suited for vertical farming or plant factory systems with artificial lighting, ensuring efficient space utilization and high productivity per unit area [12].

Rapid urbanization and the progressive limitations of arable land have accelerated interest in alternative agricultural systems that can operate independently of conventional soil-based production, including soilless cultivation techniques in greenhouses and climate controlled production systems that enhance resource use efficiency and production stability [13].In parallel with these greenhouses, more advanced systems such as indoor vertical farming systems commonly referred to as plant factories with artificial lighting or vertically structured indoor farms represent highly controlled production environments. These systems enable precise regulation of key environmental parameters, including temperature, relative humidity, light intensity and spectrum, as well as CO₂ concentration, thereby substantially reducing dependence on external climatic conditions and enhancing production stability and efficiency [14].Among these controlled environmental factors, the replacement of natural sunlight with artificial lighting represents one of the most distinctive and influential components of indoor vertical farming systems, enabling precise manipulation of light quality, intensity, and photoperiod.

Light is a fundamental source of energy and a key environmental signal for plants, regulating their photosynthesis, growth, development, and morphology. Plant structure and biomass accumulation are closely linked to light intensity, as both insufficient and excessive light levels can adversely affect plant development [15]. Light is one of the most influential environmental parameters affecting the growth and physiology of microgreens [1], as it not only regulates plant development but also influences phytochemical composition and nutritional quality, which is particularly important in microgreens due to their tendency to accumulate higher levels of bioactive compounds compared to mature plants [16].

The spectral quality of light, along with light quantity (irradiance) and photoperiod (duration of exposure), is among the most critical factors affecting plant metabolic activity, nutrient composition, and bioactive compound synthesis [13,17]. Specific wavelengths of LED light have been reported to modulate plant defense responses, enhance stress tolerance, improve the accumulation of minerals and essential nutrients, stimulate the biosynthesis of secondary metabolites, and extend shelf life while delaying senescence [18–22]. The use of LEDs as artificial light sources for plant cultivation has been widely studied, highlighting their efficiency and advantages in controlled environment agriculture [13,23,24]. Various lighting conditions have been shown to significantly influence the growth, quality, nutritional content, and medicinal value of microgreens by affecting the concentration of essential nutrients and bioactive compounds [25–31].

Recent studies have emphasized that the spectral composition of light, as well as growing conditions, plays a crucial role in regulating the biosynthesis of these secondary metabolites [32–34]. For example, in basil microgreens UV-B light (311 nm) was found to enhance polyphenolic compounds, ascorbic acid, and total antioxidant capacity without negatively affecting the photosynthetic system, whereas UV-C light (254 nm) suppressed photosynthesis and limited biomass accumulation but still induced phenolic compound accumulation and CHS activity only in the green-leaf cultivar (SL), with no positive effects observed in the purple-leaf cultivar (DO) [35]. Moreover, Thai basil cultivars demonstrated varying responses in phenolic content and antioxidant capacity under different LED treatments [36]. Different light spectra have been shown to influence biomass accumulation and essential oil production in basil, including alterations in the concentrations of key oil constituents [37]. Moreover, narrow band LED applications particularly blue, red, and their combinations have been associated with enhancements in total phenolic content, antioxidant capacity, and essential oil composition, without compromising sensory quality [7]. Additionally, light quality has been shown to influence the accumulation of specific bioactive compounds, such as methyl eugenol and β-caryophyllene, as well as mineral content and postharvest characteristics. Also, UV-A radiation has been reported to modulate phenolic and anthocyanin content, ascorbate, and tocopherol levels [38]. Blue light intensity has been linked to changes in antioxidant compounds and flavonoids, while red and far-red light supplementation has shown potential to enhance antioxidant activity [39,40]. These findings underscore the importance of selecting the appropriate light spectrum to optimize the nutritional quality of basil microgreens.

Light quality is known to regulate plant growth and development through distinct physiological and photomorphogenic mechanisms [12,13,15,16]. Red light (around 660 nm) is highly efficient in driving photosynthesis [41,42], whereas blue light (around 450 nm) plays a critical role in stomatal regulation, chlorophyll synthesis, and compact plant morphology [41,43,44]. Therefore, red–blue (R:B) combinations are commonly used to optimize biomass production and morphological traits [16,45,46]. In contrast, full-spectrum light provides a broader range of wavelengths that may better mimic natural sunlight and support balanced plant development [47,48]. The inclusion of far-red (FR, ~ 730 nm) can further influence plant architecture by triggering shade avoidance responses, potentially promoting stem elongation and biomass allocation [44,49,50]. Based on these mechanisms, it was hypothesized that different LED spectral compositions would differentially affect growth, yield, and quality parameters of basil microgreens. Previous studies have demonstrated that light intensity and spectral composition play a key role in regulating growth and the accumulation of bioactive compounds in microgreens. In particular, balanced or blue-enriched red–blue spectra have been reported to enhance biomass production and phytochemical content [51].

Red and blue LED spectra have been widely investigated in controlled environment cultivation of fully developed mature basil plant due to their pronounced effects on plant growth, photosynthetic performance, antioxidant capacity, and volatile compound composition [16,21,22,27,36]. Nevertheless, the optimal balance among red, blue, and far-red wavelengths particularly for basil microgreens and their comparative performance relative to conventional PAR-based lighting systems remain inadequately characterized. In this context, important gaps persist in understanding how complex LED spectral combinations influence basil microgreens. Although a growing body of research has addressed the effects of light quality on plant secondary metabolism, studies evaluating the combined effects of multiple spectral components including red, blue, white, and far-red wavelengths are still limited. Moreover, most existing research has predominantly focused on single wavelengths or simple red blue combinations, often conducted on mature plants rather than at the microgreen stage [52–55].

Therefore, this study aims to address this gap by evaluating the integrated effects of complex LED spectra on growth, nutritional composition, and bioactive compounds of basil microgreens under indoor vertical farming conditions. We hypothesize that red and blue light predominantly regulate photosynthetic activity and morphological development, while the inclusion of far-red wavelengths may further modulate plant architecture and biomass allocation through phytochrome-mediated responses. Full-spectrum lighting representing the PAR range was included in this study as a commonly used spectral condition for comparison, providing a baseline for evaluating the effects of different LED spectral combinations. Studies on LED spectra have been conducted, particularly in mature basil plants; however, research on basil microgreens remains relatively limited. While LED 1 and LED 2 represent commonly used spectral combinations, LED 3 includes a more complex spectrum incorporating far-red and white wavelengths, thereby providing additional insight into the effects of extended spectral composition. Furthermore, limited information is available on the interactions between blue–red spectra incorporating far-red and white wavelengths and how these interactions vary in basil, particularly in comparison with commonly used red–blue combinations and full-spectrum light within the 400–700 nm range (PAR). In this context, the present study provides a comparative evaluation of commonly used and modified light spectra under controlled vertical farming conditions.

## 2. Materials and methods

### 2.1. Plant material

Basil seeds were obtained from open-pollinated local standard varieties of the “Arzuman”® seed company. Basil plant species was chosen for this study due to its rich content of aromatic compounds, high essential oil content, wide range of applications, and its adaptability to various growing conditions.

### 2.2. Plant growing conditions

**Fig 1:**
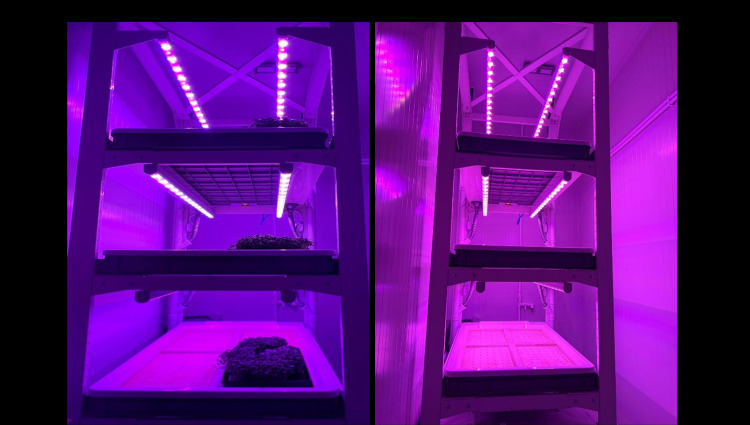
A visual representation of the controlled climate chamber used for cultivating basil microgreens.

This study was conducted in an indoor vertical climate chamber equipped with multiple layers, each illuminated by a distinct LED light spectrum, as illustrated in Fig 1. Three different LED lighting treatments were tested for the cultivation of basil microgreens. The treatments were coded as LED 1 (red–blue), LED 2 (full spectrum), and LED 3 (red–blue supplemented with white and far-red) for clarity throughout the manuscript. The following spectral combinations characterized the LED lighting treatments:

LED 1: 70% red (660 nm) and 30% blue (450 nm),

LED 2: a full-spectrum Photosynthetically Active Radiation (PAR) range of 400–700 nm, and

**Fig 2:**
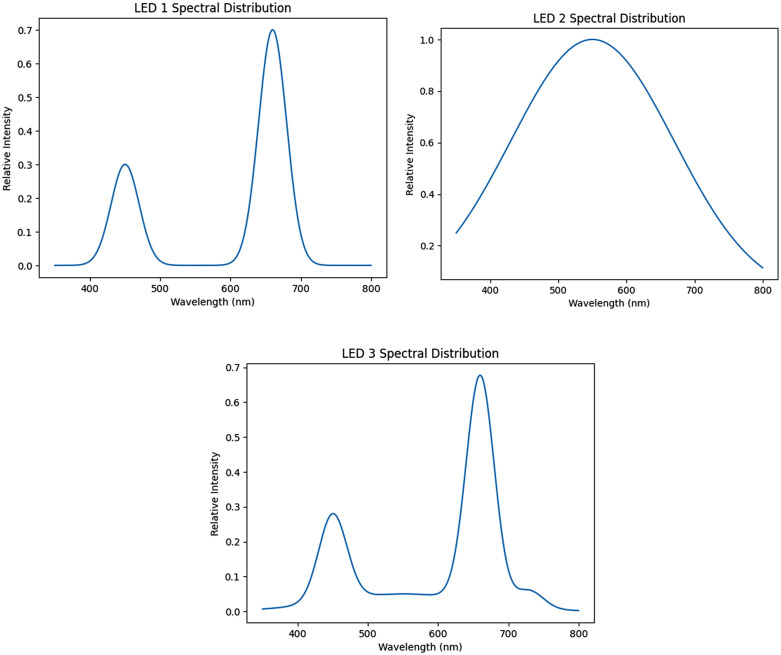
Schematic spectral distribution of the LED light treatments used in the experiment.

LED 3: a combination of 65% red (660 nm), 25% blue (450 nm), 5% white (broad spectrum, approximately 400–700 nm), and 5% far-red (730 nm) light (Fig 2).

These distinct light spectra were applied to assess their effects on the growth and physiological development of basil microgreens under controlled environmental conditions. The light intensity was maintained at 200 µmol m ⁻ ² s ⁻ ¹ photosynthetic photon flux density (PPFD) across all treatments, with a photoperiod of 16 h light and 8 h dark, corresponding to a daily light integral (DLI) of approximately 11.5 mol m ⁻ ² day ⁻ ¹. “Day and night temperatures were maintained at 24°C and 18°C, respectively, with relative humidity kept at 60%. CO₂ concentration was maintained at approximately 400 µmol mol ⁻ ¹. The growth environment was fully controlled, including stable temperature regulation, continuous air circulation (airflow ≈ 2 m s ⁻ ¹), and uniform ventilation to ensure homogeneous microclimatic conditions across all treatments.

The spectral distributions of the three LED treatments (LED 1, LED 2, and LED 3) are presented based on manufacturer-provided peak wavelengths and relative proportions of light components. LED 1 consisted of 70% red (660 nm) and 30% blue (450 nm), LED 2 represented a full-spectrum photosynthetically active radiation (PAR) range (400–700 nm), and LED 3 included 65% red (660 nm), 25% blue (450 nm), 5% white (broad spectrum), and 5% far-red (730 nm). The curves represent schematic approximations of relative spectral intensity.

In this experiment, peat was used as the growing medium. The peat-based substrate employed was Klasmann Potground P20®, characterized by a pH range of 5.5–6.5 and a high cation exchange capacity (CEC) of 150–200 meq/100 g. This substrate also included a starter fertilizer supplying essential nutrients, including nitrogen (0.8%), phosphorus (0.24%), and potassium (0.27%), to support early plant development. Basil microgreens were cultivated in transparent plastic containers measuring 16 cm × 9 cm × 7 cm (length × width × height), each filled with 250 cm³ of peat substrate. Seeds were sown at a density of 5 g m ⁻ ². The experiment was conducted with three replicates, each replicate consisting of three containers. A total of 9 containers were placed on the multilayer shelves under each LED light spectrum. Each container contained approximately 3,500 microgreen basil plants. The experiment was arranged in a completely randomized design. Measurements and analyses were performed using plant samples collected from independent replicates. For yield calculations (g m ⁻ ²), the total fresh weight of all microgreen plants harvested from each container was treated as a single replicate

### 2.3. Plant nutrition

Until the appearance of the first true leaf, irrigation was carried out using reverse osmosis (RO) filtered water only. Following this stage, a nutrient solution prepared with reverse osmosis filtered water was applied for plant irrigation. The nutrient solution was a one-quarter strength modified solution tailored for microgreen cultivation, with the following composition (mg L ⁻ ¹): N (200), P (50), K (300), Ca (200), Mg (65), Fe (5.0), Mn (0.8), Cu (0.3), Zn (0.3), B (0.3), and Mo (0.05), adjusted to pH 5.5 with an electrical conductivity (EC) of 1.2–1.6 dS cm ⁻ ¹ throughout the growth stages [3]. Subsequently, irrigation was performed as follows: 100 mL of water was applied per container on the first day, followed by 50 mL every two days until cotyledon emergence, after which 100 mL of nutrient solution per container was supplied.

### 2.4. Plant harvest

**Fig 3:**
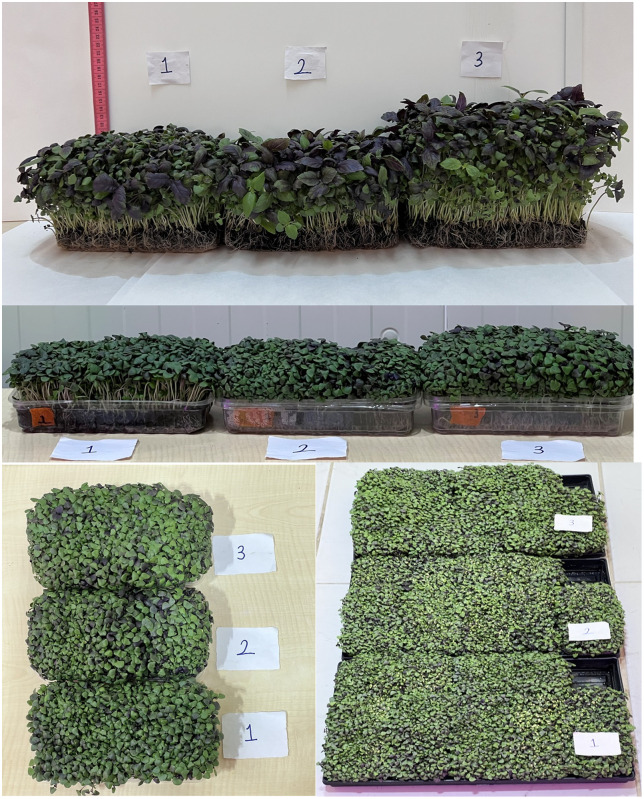
Appearance of basil microgreens grown under different LED light spectra.

Basil microgreens were cultivated for 15 days under three different LED light spectra. Harvesting was performed when the seedlings reached the stage characterized by the full development of the first true leaf, the emergence of the second true leaf, and the presence of cotyledon leaves with rounded margins [2]. No plant growth measurements were conducted at the harvest stage (Fig 3).

LED 1: 70% red (660 nm) and 30% blue (450 nm); LED 2: Full-spectrum Photosynthetically Active Radiation (PAR) within the 400–700 nm range; LED 3: 65% red (660 nm), 25% blue (450 nm), 5% white (broad spectrum around 400–700 nm), and 5% far-red (730 nm).

### 2.5. Measurements of plant growth parameters

During the harvest of basil microgreens cultivated under three different LED light spectra, plant height, hypocotyl length, stem diameter, and individual plant fresh weight were measured in four replicates. Yield per unit area (g/cm²) was calculated by harvesting all microgreens from each container, determining their fresh weight, and dividing it by the container area. Fresh weight per plant (g) was determined using a digital balance immediately after harvest. Subsequently, the samples were dried in a forced-air oven at 65 °C for 48 hours, and the dry weight per plant was recorded.

### 2.6. Color measurements of basil microgreen leaves

The luminosity (L*) and chromaticity coordinates [a* (red–green axis) and b* (blue–yellow axis)] of the harvested basil microgreen leaves were digitally measured using a handheld color spectrophotometer (HunterLab, Reston, VA, USA) [56].

### 2.7. Macro and micro-nutrient element analysis

Macro- and microelement analyses included the determination of phosphorus (P), potassium (K), magnesium (Mg), calcium (Ca), iron (Fe), manganese (Mn), copper (Cu), and zinc (Zn). Basil microgreens were harvested 15 days after seed emergence, and four replicates per treatment were used for nutrient analysis. For each replicate, approximately 5 g of fresh tissue was collected. Samples were first rinsed with distilled water and then dried in an oven at 65 °C. The dried plant material was ground using a leaf grinding mill (20-mesh), incinerated at 550 °C for 8 hours, and the resulting ash was dissolved in 3.3% (v/v) hydrochloric acid. Potassium, calcium, and magnesium concentrations were measured in emission mode, while iron, manganese, zinc, and copper were determined in absorbance mode using an atomic absorption spectrometer (AAS; Varian FS 220, Mulgrave, Victoria, Australia) [2].

### 2.8. Determination of acidity, total soluble solids (TSS), pH, and electrical conductivity (EC)

The juice extracted from basil microgreens was analyzed to determine total soluble solids (TSS), titratable acidity, pH, and electrical conductivity (EC). TSS and acidity were measured using a digital refractometer (Atago PR-101, Tokyo, Japan). Simultaneously, pH and EC values were determined with a pH/EC meter (WTW pH/Cond 3320, Weilheim, Germany) [6].

### 2.9. Determination of Total Phenols

Total phenolic content was determined using a spectrophotometric method modified from Spanos and Wrolstad [57]. For each sample, approximately 5 g of plant tissue was extracted with 80% methanol and subjected to ultrasonic treatment at 40 °C for 10 minutes to enhance the release of phenolic compounds. The extracts were then centrifuged at 1500 rpm for 5 minutes. The supernatant was mixed with Folin–Ciocalteu reagent and 20% sodium carbonate, followed by incubation in the dark for 2 hours. Absorbance was measured at 765 nm using a spectrophotometer (Analytik Jena SPECORD 200 PLUS, Jena, Germany). Total phenolic content was quantified using a gallic acid calibration curve and expressed as milligrams of gallic acid equivalents (GAE) per gram of fresh weight (FW). Analyses were conducted in four replicates using samples harvested 15 days after sowing.

### 2.10. Determination of total flavonoids

Total flavonoid content was determined according to the method described by Quettier-Deleu et al. [58]. Briefly, 1 mL of plant extract was mixed with 0.05 mL of 10% aluminum chloride (AlCl₃), 0.05 mL of 1 M potassium acetate, and 1.8 mL of Milli-Q water. The mixture was vortexed thoroughly and incubated in the dark at room temperature for 45 minutes. Following incubation, absorbance was measured at 450 nm using a spectrophotometer (Analytik Jena SPECORD 200 PLUS, Jena, Germany). Total flavonoid content was expressed as milligrams of rutin equivalents (RE) per 100 grams of fresh weight (FW). The analysis was conducted in four replicates using basil microgreens harvested 15 days after sowing.

### 2.11. Determination of Vitamin C

Vitamin C content was determined using a modified version of the method described by Elgailani et al. [59]. Basil microgreens were first processed in a juicer to obtain plant extract. Five milliliters of the extract were mixed with 45 mL of 0.4% oxalic acid solution and then filtered. From the resulting solution, 1 mL was mixed with 9 mL of 2,6-dichlorophenolindophenol reagent and vortexed thoroughly. Absorbance was measured at 518 nm using a UV–Vis spectrophotometer. The analysis was performed in four replicates using microgreens harvested 15 days after sowing.

### 2.12. Determination of Oil Content

The oil content of basil microgreens was determined using the Soxhlet extraction method, a standardized procedure for lipid quantification in plant-based samples [60]. In this study, 5 grams of dried basil microgreen samples were placed into cellulose extraction thimbles and subjected to extraction with n-hexane for 3 hours under reflux conditions using a Soxhlet apparatus. Following extraction, the samples were dried in a laboratory oven at 70 °C for 2 hours to remove residual solvent. The oil content was then determined gravimetrically and expressed as a percentage of the initial dry weight.

### 2.13. Statistical analysis

The experiment was arranged in a completely randomized design with three replicates. Each container was considered as an experimental unit. The data were subjected to analysis of variance (ANOVA) using the JMP statistical software package (version 7.0, SAS Institute Inc., 2007). When significant differences were detected at p < 0.05, treatment means were compared using the Least Significant Difference (LSD) multiple comparison test. Furthermore, a heat map and principal component analysis (PCA) were performed to evaluate the relationships among all measured variables and to identify treatment-specific patterns. The heat map was generated using the mean values of each parameter for visual comparison across treatments. PCA was conducted based on the Pearson correlation matrix, and a biplot was created to illustrate the associations between treatments and variables. Both analyses were carried out using ClustVis software version 2.0 (https://biit.cs.ut.ee/clustvis/; accessed on 12 May 2025).

This study did not require specific ethical approval or permits, as it involved cultivated plant materials and did not include protected species, endangered plants, or field sampling. All experiments were conducted under controlled conditions at Çukurova University, Türkiye, in accordance with relevant institutional and national regulations. No deviations from the originally planned study protocol were made during the course of the study.

## 3. Results

### 3.1. Effects of LED light spectra on growth and development of basil microgreens

Table 1 presents the growth parameters of basil microgreens cultivated under three different LED light spectra (LED 1, LED 2, and LED 3), including plant height, hypocotyl length, stem diameter, individual plant weight, and total yield. The results showed that the applied LED spectra had a statistically significant effect on plant height (p = 0.0001). Plants grown under LED 3 reached the greatest height (12.14 cm), followed by LED 2 (9.97 cm) and LED 1 (8.62 cm). Hypocotyl length was also significantly affected by the light treatments (p = 0.0024). The longest hypocotyls were observed under LED 3 (7.53 cm), whereas LED 1 (5.26 cm) and LED 2 (5.54 cm) did not differ significantly from each other. In contrast, stem diameter and individual single plant weight were not significantly affected by the different LED treatments. However, total yield per cultivation tray exhibited a statistically significant difference among treatments (p = 0.0388); the recorded values were 0.53 g/cm² for LED 3, 0.40 g/cm² for LED 1, and 0.37 g/cm² for LED 2. Overall, these findings suggest that the LED light spectra had a significant impact on vertical growth parameters, including plant height, hypocotyl length, and total yield.

**Table 1:** Effect of LED light treatments on growth parameters of basil microgreens.

LED treatments*	Plant Length (cm)	Hypocotyl Length (cm)	Stem Diameter (mm)	Single Plant Weight (g)	Yield (g/cm^2^)
LED 1	8.62 c	5.26 b	1.21	0.280	0.40 b
LED 2	9.97 b	5.54 b	1.29	0.330	0.37 b
LED 3	12.14 a	7.53 a	1.28	0.350	0.53 a
*p*	0.0001	0.0024	0.6427	0.244	0.0388
LSD_0.05_	1.05	1.11	NS	NS	0,12

*LED 1: 70% red (660 nm) and 30% blue (450 nm); LED 2: Full-spectrum Photosynthetically Active Radiation (PAR) within the 400–700 nm range; LED 3: 65% red (660 nm), 25% blue (450 nm), 5% white (broad spectrum around 400–700 nm), and 5% far-red (730 nm). Means followed by different letters in the same column differ significantly at *p* < 0.05 (LSD test). No significant difference was observed between means sharing the same letter within the same column (*p* < 0.05). NS: Not significant.

### 3.2. Effect of LED light spectra on brix, acidity, ph, and electrical conductivity in basil microgreens

Table 2 presents the effects of different LED light spectra on acidity, total soluble solids (Brix), pH, and electrical conductivity (EC, µS cm ⁻ ¹) of basil microgreens. The influence of the LED treatments varied across the measured parameters. For acidity, LED 3 resulted in significantly higher values (0.18) compared to LED 1 and LED 2, both of which recorded 0.12 (p = 0.0002). This indicates a strong influence of spectral composition on organic acid accumulation. In terms of Brix (%), no statistically significant differences were observed among the treatments. The pH values were measured as 6.06 for LED 1, 6.14 for LED 2, and 6.16 for LED 3. For electrical conductivity (EC), the measured values were 12,780 µS cm ⁻ ¹ for LED 1, 12,740 µS cm ⁻ ¹ for LED 2, and 13,400 µS cm ⁻ ¹ for LED 3 (p < 0.0001). In summary, the application of different LED light spectra significantly affected acidity, pH, and EC values in basil microgreens. LED 3 was particularly effective in increasing acidity and EC, whereas no statistically significant differences were observed among treatments for Brix content.

**Table 2:** Effect of LED light treatments on acidity, Brix, pH, and electrical conductivity in basil microgreens.

LED treatments*	Acidity (%)	Brix (%)	pH	EC(µS cm ^− 1^)
LED 1	0.12 b	2.62	6.06 b	12780 b
LED 2	0.12 b	2.67	6.14 a	12740 b
LED 3	0.18 a	2.55	6.16 a	13400 a
*p*	0.0002	0.1676	<.0001	<.0001
LSD_0.05_	0.02	NS	0.02	0.14

*LED 1: 70% red (660 nm) and 30% blue (450 nm); LED 2: Full-spectrum Photosynthetically Active Radiation (PAR) within the 400–700 nm range; LED 3: 65% red (660 nm), 25% blue (450 nm), 5% white (broad spectrum around 400–700 nm), and 5% far-red (730 nm). Means followed by different letters in the same column differ significantly at *p* < 0.05 (LSD test). No significant difference was observed between means sharing the same letter within the same column (*p* < 0.05). NS: Not significant.

### 3.3. Effect of LED light spectra on phenolic, flavonoid, and vitamin C contents in basil microgreens

**Fig 4:**
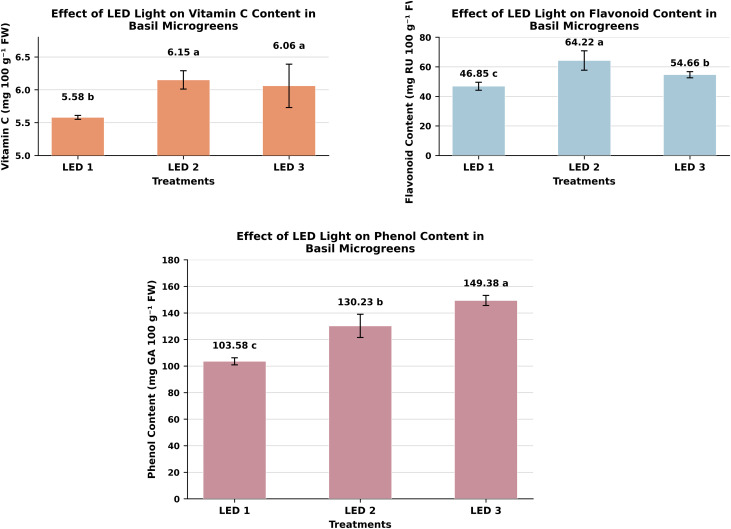
Effects of different LED light treatments on total phenolic content, total flavonoid content, and vitamin C content in basil microgreens. LED 1: 70% red (660 nm) and 30% blue (450 nm); LED 2: Full-spectrum Photosynthetically Active Radiation (PAR) within the 400–700 nm range; LED 3: 65% red (660 nm), 25% blue (450 nm), 5% white (broad spectrum around 400–700 nm), and 5% far-red (730 nm). Different letters on the histogram indicate statistically significant differences at *p* < 0.05 according to the LSD test. There is no significant difference between means with the same letter in the same color histogram section.

Fig 4 illustrates the effects of different LED light treatments on the total phenolic, flavonoid, and vitamin C contents of basil microgreens. According to statistical analysis, all three parameters were significantly affected by the light spectra applied. For total phenolic content, LED 3 resulted in the highest accumulation, with a value of 149.38 mg GA 100 g ⁻ ¹ FW (*p* < 0.0001). LED 2 followed with 130.23 mg GA 100 g ⁻ ¹ FW, while LED 1 recorded the lowest phenolic content at 103.58 mg GA 100 g ⁻ ¹ FW. These results indicate that spectral composition has pronounced effect on phenolic compound accumulation in basil microgreens. Regarding total flavonoid content, LED 2 showed the highest value (64.22 mg RU 100 g ⁻ ¹ FW), which was statistically different from both LED 3 (54.66 mg RU 100 g ⁻ ¹ FW) and LED 1 (46.85 mg RU 100 g ⁻ ¹ FW) (*p* = 0.0009). These findings suggest that flavonoid biosynthesis is responsive to variations in LED light spectra.

For vitamin C content, no statistically significant difference was observed between the LED 2 and LED 3 treatments; however, both resulted in higher values (6.15 and 6.06 mg 100 g ⁻ ¹ FW, respectively) compared to LED 1, which recorded the lowest content at 5.58 mg 100 g ⁻ ¹ FW (*p* = 0.0080). In summary, LED light spectra significantly influenced the accumulation of phenolic compounds, flavonoids, and vitamin C in basil microgreens. While LED 3 was most effective in enhancing total phenolic content, LED 2 led to the highest flavonoid and vitamin C concentrations.

### 3.4. Effect of LED light spectra on oil content, dry matter, and color parameters (L, a, b) in basil microgreens

**Fig 5:**
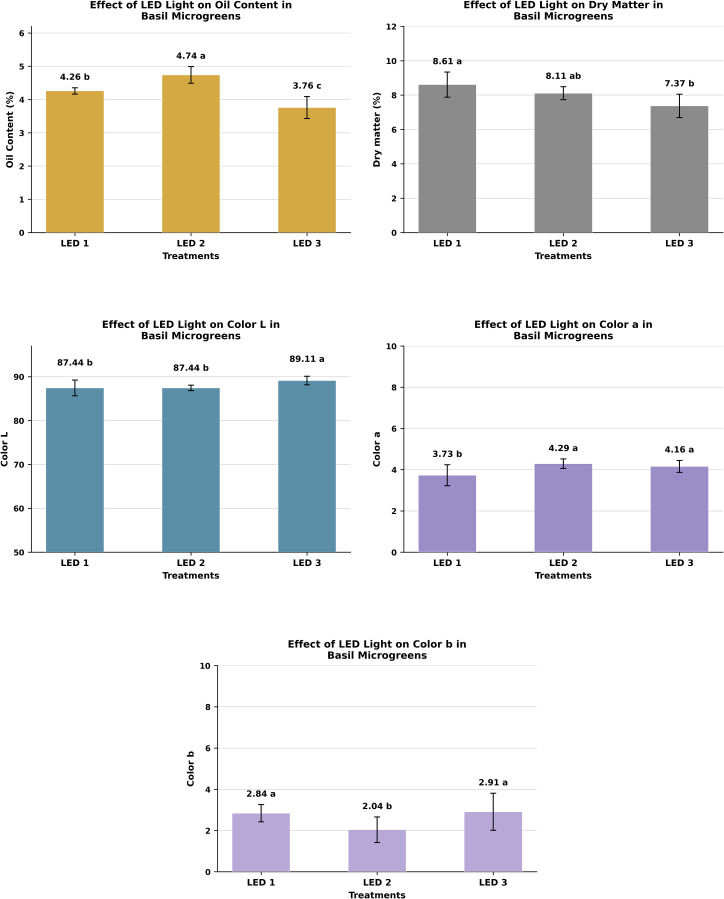
The effects of different LED light applications on oil content, dry weight, and color (L*, a*, b*) of the basil microgreens. LED **1:** 70% red (660 nm) and 30% blue (450 nm); LED 2: Full-spectrum Photosynthetically Active Radiation (PAR) within the 400–700 nm range; LED 3: 65% red (660 nm), 25% blue (450 nm), 5% white (broad spectrum around 400–700 nm), and 5% far-red (730 nm). Different letters on the histogram indicate statistically significant differences at *p* < 0.05 according to the LSD test. There is no significant difference between means with the same letter in the same color histogram section.

In this study, the impact of different LED light spectra on oil content, dry matter percentage, and color attributes (L*, a*, b*) of basil microgreens was evaluated. The results revealed statistically significant differences among treatments for several parameters (Fig 5). For oil content, LED 2 yielded the highest value (4.74%), which was significantly greater than that of LED 1 (4.26%) and LED 3 (3.76%) (p = 0.0011). These findings indicate that LED light quality has a considerable influence on oil accumulation, with LED 2 being the most effective in promoting oil synthesis. Regarding dry matter content, LED 1 recorded the highest value (8.61%), followed by LED 2 (8.11%) and LED 3 (7.37%). For color parameters, a difference was observed in the L value* (*p* = 0.0184), with LED 3 producing the highest lightness (89.11), while LED 1 and LED 2 showed equal values (87.44). No significant differences were found for a* (*p* = 0.1219) or b* (*p* = 0.1821) values. Nonetheless, LED 2 yielded the highest a* value (4.29), and LED 3 exhibited the highest b* value (2.91), indicating slight variation in red–green and yellow–blue color tones, respectively. LED light treatments significantly influenced oil content and color brightness (L*) in basil microgreens. LED 2 was the most effective in enhancing oil content, whereas LED 3 improved visual brightness. These results demonstrate the potential of targeted LED spectra to modulate key quality attributes in microgreen production.

### 3.5. Effects of different LED light treatments on magnesium, potassium, and calcium content in basil microgreens

The effects of different LED light treatments on the accumulation of magnesium (Mg), potassium (K), and calcium (Ca) in basil microgreens are summarized in Table 3. The highest measured concentrations were 27.64 mg for magnesium (LED 1), 292.69 mg for potassium (LED 1), and 34.75 mg for calcium (LED 1). However, the differences among the LED treatments were not statistically significant (p > 0.05), indicating that LED spectral composition did not have a significant effect on the accumulation of these macronutrients under the conditions of this study.

**Table 3:** Effect of LED light treatments on magnesium (Mg), potassium (K), and calcium (Ca) contents (mg/100 g FW) in basil microgreens.

LED treatments*	Mg	K	Ca
LED 1	27.64	292.69	34.75
LED 2	26.94	278.64	30.87
LED 3	27.25	284.13	29.07
*p*	0.9793	0.2368	0.5142
LSD_0.05_	NS	NS	NS

*LED 1: 70% red (660 nm) and 30% blue (450 nm); LED 2: Full-spectrum Photosynthetically Active Radiation (PAR) within the 400–700 nm range; LED 3: 65% red (660 nm), 25% blue (450 nm), 5% white (broad spectrum around 400–700 nm), and 5% far-red (730 nm). Means followed by different letters in the same column differ significantly at *p* < 0.05 (LSD test). No significant difference was observed between means sharing the same letter within the same column (*p* < 0.05). NS: Not significant.

### 3.6. Effects of different LED light treatments on copper, manganese, iron, and zinc content in basil microgreens

Table 4 summarizes the effects of different LED light treatments on the micronutrient content of basil microgreens. LED 1 resulted in the highest concentrations of copper (Cu, 34.20 mg 100 g ⁻ ¹ FW) and iron (Fe, 984.96 mg), which were significantly affected by the light treatments (Cu: p = 0.0040; Fe: p < 0.0001). In contrast, manganese (Mn) and zinc (Zn) contents were not significantly influenced by the LED spectra (p > 0.05). These results indicate that, under the conditions of this study, LED 1 was most effective in enhancing Cu and Fe accumulation, while Mn and Zn levels remained statistically unchanged.

**Table 4:** Effect of LED light treatments on copper (Cu), manganese (Mn), iron (Fe), and zinc (Zn) contents (µg 100 g ⁻ ¹ FW) in basil microgreens.

LED treatments*	Cu	Mn	Fe	Zn
LED 1	34.20 a	340.86	984.96 a	365.94 a
LED 2	15.96 b	269.04	735.30 b	310.08 ab
LED 3	13.84 b	278.16	647.49 b	286.14 b
*p*	0.0040*	0.4577	<.0001	0.118
LSD_0.05_	10.87	NS	98.18	NS

*LED 1: 70% red (660 nm) and 30% blue (450 nm); LED 2: Full-spectrum Photosynthetically Active Radiation (PAR) within the 400–700 nm range; LED 3: 65% red (660 nm), 25% blue (450 nm), 5% white (broad spectrum around 400–700 nm), and 5% far-red (730 nm). Means followed by different letters in the same column differ significantly at *p* < 0.05 (LSD test). No significant difference was observed between means sharing the same letter within the same column (*p* < 0.05). NS: Not significant.

### 3.7. Multivariate analysis of morphological, physiological, and biochemical responses under LED light treatments

**Fig 6:**
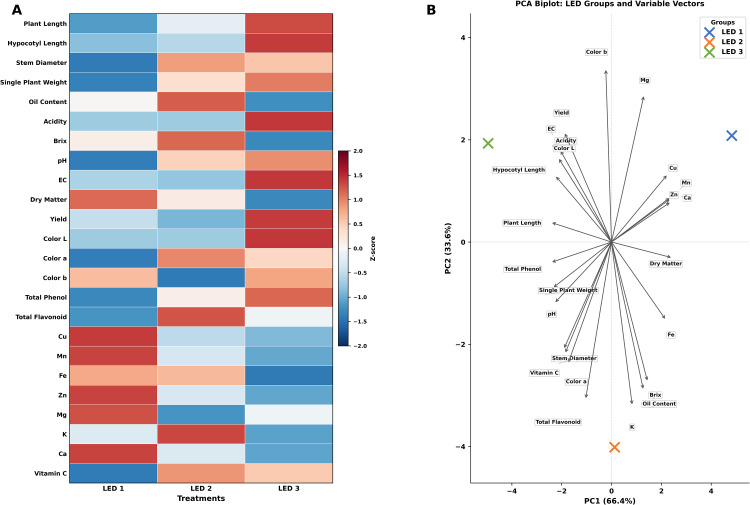
Heat map (A) and PCA (B) biplot illustrating the effects of LED light treatments on morphological, physiological, and biochemical traits of basil microgreens. The heat map shows treatment-related variations, while the PCA biplot highlights associations between variables and treatments. *LED 1: 70% red (660 nm) and 30% blue (450 nm); LED 2: Full-spectrum Photosynthetically Active Radiation (PAR) within the 400–700 nm range; LED 3: 65% red (660 nm), 25% blue (450 nm), 5% white (broad spectrum around 400–700 nm), and 5% far-red (730 nm).

Principal Component Analysis (PCA) was performed to explore the relationships among 24 physiological and biochemical traits under different LED light treatments (Fig 6). The first two principal components (PC1 and PC2) together explained a substantial portion of the total variation among the treatments (PC1: 66.4%, PC2: 33.6%; actual values to be filled). The PCA biplot separated the LED treatments based on their distinct influence on plant traits. LED 3 was positioned in the positive region of both PC1 and PC2, indicating a strong association with traits such as hypocotyl length, plant length, single plant weight, color L, color b, EC, and total phenolic content. This clustering suggests that LED 3 is particularly effective in enhancing both vegetative growth and visual quality parameters of the microgreens. LED 2, located in the negative PC2 and slightly positive PC1 region, showed stronger associations with oil content, Brix, potassium (K), total flavonoids, and vitamin C, indicating its effectiveness in promoting biochemical quality and nutritional content. In contrast, LED 1 clustered on the negative side of PC1, aligning closely with mineral nutrients such as Ca, Mg, Cu, Mn, and Zn, as well as dry matter and Fe, reflecting its influence on nutrient accumulation and structural integrity. The directions and lengths of the red arrows (variable vectors) indicate the magnitude and contribution of each parameter to the principal components. Traits such as plant length, EC, and phenolic content contributed strongly to PC1, whereas oil content, total flavonoids, and color traits had more influence on PC2. Parameters pointing in similar directions are positively correlated, while those pointing in opposite directions are negatively associated.

In the heat map graph (Fig 6), among the three light treatments, LED 3 stood out by strongly enhancing key productivity and visual quality parameters such as yield, hypocotyl length, EC, and color L, all indicated by deep red tones. Additionally, total phenolic content, single plant weight, and color b showed moderate increases (light red), reflecting their positive effects on both morphological development and visual appeal. However, total flavonoid and Mg content remained neutral (white), and certain micronutrients such as Mn and Zn were at their lowest levels (blue). In contrast, LED 2 strongly enhanced potassium (K), oil content, Brix, and total flavonoids (dark red), with moderate increases in color a, stem diameter, and vitamin C, indicating its benefit for sweetness and antioxidant-related traits. Meanwhile, LED 1 was most effective in improving mineral accumulation, particularly Mg, Cu, Ca, Mn, and Zn (dark red), and also led to moderate increases in dry matter and Fe content, suggesting its contribution to nutritional density.

## 4. Discussion

In basil microgreen cultivation, plant quality parameters and yield performance are key determinants of production efficiency and the nutritional value of the final product. One of the key advantages of LED technology is its ability to deliver targeted light wavelengths that can modulate plant morphology and enhance phytochemical composition [10,13,32]. Light quality particularly that provided by LED lighting systems plays a central role in enhancing photosynthetic efficiency, regulating morphological development, and stimulating the biosynthesis of bioactive compounds [32]. Therefore, the selection of an appropriate LED light spectrum directly influences both yield and quality. Generating new scientific insights into the effects of different LED spectra is essential for optimizing microgreen production systems. Identifying the most suitable light conditions can offer significant advantages to producers by enabling higher yields and improved crop quality.

### 4.1. Effects of LED light spectra on morphological traits and biomass accumulation in basil microgreens

The current study demonstrated that different LED light spectra significantly influenced plant growth parameters in basil microgreens. Among the treatments, LED 3 resulted in superior outcomes in terms of plant height and hypocotyl elongation, indicating a positive effect on overall vegetative development. These findings highlight the importance of spectral composition in supporting optimal plant growth, reinforcing the notion that light functions not only as an energy source but also as a key environmental signal [61]. While red light is widely recognized for its role in driving photosynthesis, additional wavelengths such as blue and far-red are also essential. Blue light regulates stomatal aperture, thereby enhancing CO₂ uptake and transpiration, and mitigates the risk of “red light syndrome,” which can impair plant morphology and alter gene expression patterns [62].

Previous studies support these observations. For instance, Teliban et al. [63], reported that varying light conditions induced differences in both phenotypic traits and biochemical responses. In their experiment, changes in biomass accumulation were relatively modest among light treatments, though trends were observable. Skowron et al. [35], found that basil microgreens of the ‘Sweet Large’ and ‘Dark Opal’ cultivars grown under an RGB spectrum supplemented with UV-B exhibited significant gains in biomass. Fresh mass increased by 52% and 32%, while dry mass rose by 49% and 43% in the respective cultivars. These results are consistent with the current findings and those of Rahman et al. [52], who reported enhanced fresh biomass under blue plus far-red (B + FR) light combinations in sweet basil. Similarly, Craver et al. [64] and Brazaitytė et al. [65] demonstrated that tailored light supplementation particularly red-blue combinations increased yield and promoted hypocotyl elongation, paralleling the trends observed in the present study.

### 4.2. Effect of LED light spectra on brix, acidity, ph, and electrical conductivity in basil microgreens

In this study, analysis of acidity, pH, °Brix, and electrical conductivity (EC) revealed that LED 3 resulted in the highest acidity, pH, and EC values among treatments. However, differences in °Brix levels were not statistically significant. LED 1 and LED 2 produced comparable values for these traits. These findings are partially in line with those of Mahajan et al. [66], who reported that a 70:30 red-to-blue light ratio led to the highest °Brix value (6.17%) in radish, suggesting that specific spectral combinations can influence sugar accumulation. Similarly, Bantis [16], observed that total soluble solids (TSS) in microgreens varied depending on light spectrum and plant species. For example, mustard, radish, borage, and green basil showed the lowest TSS under a red/blue ratio of 2, while red amaranth and pea shoots responded differently under varying light conditions.

### 4.3. Effect of LED light spectra on phenolic, flavonoid, and vitamin C contents in basil microgreens

Light quality is a key environmental signal that regulates the biosynthesis of secondary metabolites [67]. In the present study, LED light treatments significantly influenced the accumulation of phenolic and flavonoid compounds, while changes in vitamin C levels were more moderate. Sobhanan et al. [68] reported that, in radish microgreens, the application of a blue–red–far-red spectrum at 300 µmol m ⁻ ² s ⁻ ¹ with a 20 h photoperiod resulted in the highest total phenolic content (355.77 ± 8.42 mg GAE 100 g ⁻ ¹), representing a 59% increase compared to the control. This highlights the role of spectral composition in stimulating the production of bioactive compounds such as antioxidants. Lobiuc et al. [69] reported that increasing the proportion of red light enhanced phenolic content in green basil, consistent with the cultivar used in this study. Similarly, Samuolienė et al. [70] found that Romaine lettuce grown under supplemental red light (peak 622 nm) had elevated phenolic content. However, anthocyanin and tocopherol levels were lower under narrower light peaks (595 nm). Plants typically increase phenolic synthesis as a defense mechanism in response to abiotic and biotic stress, helping to protect the photosynthetic apparatus [71]. Giménez et al. [72] also observed that red-blue LED lighting increased total phenolic content in purslane microgreens compared to fluorescent and far-red combinations. In another study, Narouei et al. [7] demonstrated that narrow-bandwidth LEDs (blue, red, and red-blue) enhanced total phenolic content and anti-radical activity in basil microgreens compared to sunlight and white light, further supporting the light-quality-dependent regulation of phytochemicals.

### 4.4. Effect of LED light spectra on oil content, dry matter, and color parameters (L, a, b) in basil microgreens

Additionally, our results showed that LED 2 significantly enhanced oil content in basil microgreens, suggesting that specific spectral compositions may stimulate lipid biosynthesis and improve the nutritional and commercial attributes of the crop. Although differences in dry matter accumulation were observed across treatments, they were not statistically significant, indicating the need for further investigation. Variations in color parameters, particularly brightness (L*), also revealed that LED spectra can influence the visual appeal of microgreens, an essential factor for consumer acceptance and marketability. In line with our findings, Fayezizadeh et al. [73] emphasized the importance of genetic variation in determining the color characteristics of basil microgreens, including differences in chlorophyll and anthocyanin content. That study showed notable inter-cultivar variation in terms of color brightness and saturation, with some cultivars displaying more vibrant and intense pigmentation than others, thus underlining the interplay between genotype and light quality in shaping the visual attributes of microgreens.

Dry matter content is an essential indicator of biomass accumulation and overall plant health in microgreen production. Although LED 1 resulted in the highest dry matter content in this study, the differences among treatments were not statistically significant. Similar findings have been reported in previous studies, suggesting that while light quality can influence water content and tissue density, its effects on dry matter accumulation may be subtle or cultivar-dependent [12,45,74]. The absence of statistical significance in dry matter variation may also be related to the short cultivation period typical of microgreens, during which biomass partitioning is more influenced by water retention than structural compounds.

The increase in oil content under LED 2, which provides full-spectrum photosynthetically active radiation (PAR, 400–700 nm), may be associated with the broad wavelength range supporting multiple photoreceptors involved in lipid biosynthesis. Full-spectrum lighting mimics natural sunlight and may stimulate both photosynthetic activity and secondary metabolic pathways, including lipid accumulation. This result aligns with previous reports indicating that broader spectral compositions can enhance oil synthesis in leafy vegetables. In basil, different light spectra influence not only the essential oil yield and the concentration of its major constituents, but the genotype also significantly affects oil composition, suggesting that both cultivar and light quality are essential factors [37,75].

The present study demonstrated that LED light treatments significantly influenced the brightness (L*) of basil microgreens, while the red–green (a*) and yellow–blue (b*) color parameters remained statistically unaffected. Specifically, LED 3 resulted in the highest L* value (89.11), suggesting enhanced visual brightness and potentially improved market appeal. This finding is noteworthy from a consumer perception standpoint, as improved visual quality can enhance marketability. Samuolienė et al. [40] reported that tailored LED lighting, particularly blue (455 and 470 nm) and green (505 nm) wavelengths, enhanced anthocyanin content in red leaf lettuce, thereby improving pigmentation and visual quality. These findings highlight the importance of optimizing light spectra not only to support plant growth and biochemical composition but also to enhance visual attributes that are critical for consumer appeal and marketability. Consistent with these observations, Flores et al. [76] evaluated the impact of different light intensities on the color characteristics of Brassicaceae microgreens. Their results indicated that specific light intensities significantly influenced L*, a*, and b* values, further supporting the notion that light quality and intensity can be strategically manipulated to enhance the visual and nutritional quality of microgreens.

### 4.5. Effects of LED light spectra on mineral nutrient contents of basil microgreens

This study demonstrated that LED light treatments did not result in significant differences in the concentrations of Mg, K, and Ca in plant tissues. These findings suggest that although LED lighting can modulate various physiological and biochemical processes in plants, its influence on the accumulation of these essential mineral nutrients appears to be limited. This indicates that other factors, such as nutrient availability in the growing medium and species-specific metabolic pathways, may play a more dominant role in regulating Mg, K, and Ca uptake and accumulation.

Nonetheless, previous studies have reported differing outcomes depending on light spectral composition. For example, Mahajan et al. [66] reported a significant increase in potassium accumulation in mustard, lettuce, radish, and broccoli grown under a red:blue (70:30) LED light ratio compared to white light. These findings support the notion that specific light spectra can enhance nutrient uptake in particular species. Similarly, Narouei et al. [7] found that microgreens exposed to blue LED lighting exhibited significantly higher potassium levels and superior visual quality compared to mature plants and those treated with other light spectra.

In this study, LED light treatments did not lead to significant changes in the concentrations of Mn and Zn, except for Cu and Fe. These results suggest that the influence of LED light spectra on micronutrient accumulation in plants may be element-specific. Notably, the significant variation observed in Cu and Fe levels implies that copper may be more responsive to changes in light quality. In contrast, the lack of significant differences in Mn and Zn concentrations indicates that their uptake and translocation are likely influenced not only by light spectrum but also by other factors, such as the composition of the nutrient solution, species-specific physiological mechanisms, and broader environmental conditions. Supporting this interpretation, Balık et al. [2] investigated the impact of different growing environments on the macro- and microelement composition of microgreens and reported significant variability in nutrient levels among plant species. These differences were attributed to suboptimal growing conditions and stress-induced physiological responses. Their findings align with those of the present study, highlighting the importance of environmental factors in shaping the nutritional profile of microgreens.

Furthermore, literature reports have demonstrated that specific wavelengths and intensities within the light spectrum can enhance mineral nutrient accumulation in microgreens such as mustard and kale. For instance, blue light has been shown to activate phototropin, a blue light receptor that promotes the opening of ion channels on the plasma membrane, thereby facilitating ion transport and nutrient uptake [77,78]. Several studies have confirmed the positive effects of blue light on mineral accumulation across different microgreen species [79,80].

Additionally, several studies have demonstrated that red light can enhance the mineral nutrient content of specific microgreen species, particularly buckwheat and beet [81,82]. These findings suggest that the effects of different light wavelengths on nutrient uptake and accumulation may be species-specific. The present study reinforces the beneficial role of light quality in improving the nutritional composition of microgreens, underscoring the importance of optimizing light conditions to maximize their mineral content.

Future research should aim to elucidate the underlying physiological and molecular mechanisms that govern light-induced nutrient accumulation. Furthermore, exploring the practical implementation of these insights in controlled environment agriculture may facilitate the development of efficient cultivation strategies for producing high-quality, nutrient-dense microgreens at a commercial scale.

### 4.6. Multivariate assessment of plant responses to varying LED light spectra: insights into morphological, physiological, and biochemical traits

The combined heat map and PCA analyses provided a comprehensive understanding of how different LED light treatments influenced the physiological and biochemical responses of basil microgreens. LED 3 was particularly effective in enhancing growth and appearance related traits, including yield, hypocotyl length, electrical conductivity (EC), and brightness (color L), as indicated by its clustering with these variables in the PCA biplot and deep red shading in the heat map. Moderate increases in total phenolic content, single plant weight, and color b further supported the visual and nutritional benefits of LED 3. Conversely, LED 2 was more closely associated with oil content, Brix, potassium, and total flavonoids, suggesting its potential to enhance taste and antioxidant capacity. LED 1 aligned more with mineral elements such as Mg, Ca, Cu, and Zn, indicating its role in promoting nutrient accumulation. These findings reveal that the spectral composition of light can differentially influence growth and quality traits in cultivated basil microgreens, with LED 3 exhibiting the most balanced and favorable overall performance.

## 5. Conclusions

This study highlights the critical role of LED light spectral composition in shaping the growth performance and phytochemical quality of basil microgreens under indoor vertical farming conditions. Among the tested light treatments, LED 3 comprising red, blue, white, and far-red wavelengths was most effective in enhancing morphological parameters such as plant height, hypocotyl length, and yield, as well as improving visual brightness (L*), acidity, electrical conductivity, and total phenolic content. In contrast, LED 2, which provided full spectrum PAR light, significantly promoted flavonoid accumulation, vitamin C content, and oil synthesis. LED 1, with a high red to blue ratio, yielded the highest dry matter and copper concentrations, though it had a more limited effect on other quality traits.

The findings demonstrate that spectral quality can be strategically manipulated not only to enhance biomass and phytochemical accumulation but also to improve visual traits important for consumer appeal and marketability. Although macro and micro nutrient accumulation showed limited responsiveness to light treatments, except for copper and iron, this suggests that mineral uptake may be more dependent on species-specific physiology and substrate conditions than light composition alone.

In conclusion, the use of tailored LED light spectra offers a valuable approach to optimize both the nutritional value and aesthetic quality of basil microgreens, supporting sustainable production practices in controlled environment agriculture systems such as indoor vertical farms. LED 3 is recommended as an optimal light source for producing high-quality basil microgreens. It emerges as the most favorable treatment when prioritizing yield, biomass, and quality attributes of microgreen basil, making it a promising option for commercial microgreen production under controlled environments. Future studies should specifically investigate the interactions between light spectra, cultivar traits, and environmental factors, in order to provide clearer insights and refine lighting strategies for commercial microgreen cultivation.
