# Supplementary material for: Effects of LED spectral compositions on yield, growth, and nutritional quality of basil microgreens in indoor vertical farming
Source: PLoS One. 2026 Jul 7;21(7):e0352317. doi: 10.1371/journal.pone.0352317 (PMC13340805; doi:10.1371/journal.pone.0352317)
Supplement: Attachment — Submitted filename: Response to reviewers April 7, 2026.docx [file pone.0352317.s003.docx]

Dear Editor and Reviewers,

We would like to express our sincere gratitude for your constructive comments, and insightful suggestions, which have significantly contributed to improving the quality of our manuscript. All comments have been carefully considered and thoroughly addressed. We have provided detailed, point-by-point responses to each comment below. In addition, all revisions in the manuscript have been clearly indicated using track changes and highlighted in blue for ease of evaluation.

In accordance with the journal’s request, we have included a complete copy of the “Inclusivity in Global Research” questionnaire and uploaded it as a separate Supporting Information file during resubmission. In addition, all figures have been removed from the main manuscript file, and each figure has been uploaded separately to the system in TIFF format, as required.

**EDITOR COMMENTS**

Comment 1. The authors are advised to put additional efforts in meticulously addressing several major omissions listed in the reviewers' comments. Reviewer #2 and Reviewer #3 encouraged authors to more clearly articulate the specific research gap this study fills and to address the mechanistic hypothesis, i.e., what arethe expected advances of red/blues light over the full light spectrum as well as possible advances of involvement/skipping the far-red wavelengths. Moreover, Reviewer #2 suggested to explain how the experimental design or findings differ meaningfully from prior studies, and the unique practical or scientific contribution of the work.

Response 1. We thank the reviewers for these valuable and constructive suggestions. In response, we have revised the Introduction section to better clarify the research gap, underlying mechanistic hypothesis, and the expected advantages of different light spectra, including the role of far-red wavelengths. In addition, the novelty and the practical and scientific contributions of the study have been more clearly articulated. These revisions have been incorporated in the final three paragraphs of the Introduction (highlighted in blue).

Comment 2. Statistical testing information is missing and the existence of experimental replications is questionable.

Response 2: In response to the editor’s comment, the following information has been added to the Materials and Methods section and is highlighted in blue: “The experiment was conducted with three replicates, each replicate consisting of three containers. A total of 9 containers were placed on the multilayer shelves under each LED light spectrum. Each container contained approximately 3,500 microgreen basil plants. The experiment was arranged in a completely randomized design. Measurements and analyses were performed using plant samples collected from independent replicates. For yield calculations (g m⁻²), the total fresh weight of all microgreen plants harvested from each container was treated as a single replicate.”

Comment 3. Finally, the authors are advised not to overestimate their findings whenpresenting conclusions of the study. Using the expressions such as "fruit quality" and similar implies toogenerative content.

Response 3: We thank the reviewer for this valuable comment. We have carefully considered this point and revised the manuscript accordingly by moderating our statements and avoiding overly general expressions such as ‘fruit quality,’ ensuring that the conclusions are presented in a more precise and scientifically accurate manner.

**RESPONSES TO REVIEWER 3 COMMENTS**

Reviewer #3: This manuscript describes the effects of 3 light spectra on growthand quality parameters of microgreen basil in a 2 week experiment. The manuscript has serious flaws:

Comment 1. It is mentioned that ANOVA is used, however, statistical information ismissing. Manuscript gives the impression that there have been no independentrepetitions. Line 112: 3 layers each illuminated by a different light spectrum.It could easily occur that the top layer has a somewhat higher or lowertemperature, and without proper randomization and repetition a temperatureeffect would be wrongly seen as a light spectrum effect.

Response 1. We thank the reviewer for this valuable comment. We have revised the Materials and Methods section to clearly describe the experimental design and replication. The experiment was conducted in a completely randomized design with three independent replicates, and each container was defined as an experimental unit. Relevant details regarding replication and sampling procedures have been added and highlighted in blue. The statistical analysis, including ANOVA and LSD test (p < 0.05), was already described in the manuscript. These revisions aim to clarify the independence of replicates and the statistical approach used.

We thank the reviewer for this important observation. The vertical farming system used in this study was operated under controlled environmental conditions, with a well-established air circulation and climate control system ensuring uniform temperature distribution across all layers. Continuous air movement and regulated ventilation minimized the possibility of temperature gradients between the upper and lower shelves. Therefore, potential temperature differences among layers were considered negligible. In addition, the experimental design and replication structure have been clearly described in the revised manuscript to ensure that the observed effects can be attributed to the light spectrum treatments rather than environmental variation.

Comment 2. Only 3 light spectra are compared but there is no logic provided, and thefigures of the spectra are not given (a full spectrum within 400-700 nm is notenough information).

Response 2. We thank the reviewer for this valuable comment. In response, the spectral distribution graphs of each LED treatment have been added to the revised manuscript as Figure 2. These figures provide a clear representation of the light spectra based on the manufacturer-provided peak wavelengths and relative proportions of the spectral components. The relevant additions have been incorporated into the manuscript and highlighted in blue.

Comment 3. There is no mechanistic hypothesis, e.g. what would be expected from onlyR:B versus full spectrum and why? Or from a full spectrum with or without FRand why? The hypothesis given is merely: we expect effects of LED spectrum onthe variables we measure.

Response 3. We thank the reviewer for this insightful comment. We agree that the initial version of the manuscript did not sufficiently present the mechanistic basis of the hypothesis. In response, the Introduction section has been revised to clearly describe the expected physiological and photomorphogenic effects of red–blue light, full-spectrum light, and far-red inclusion. The revised text now explains the underlying mechanisms and the rationale for comparing these light treatments. These additions have been incorporated into the last three paragraphs of the Introduction in the revised manuscript and are highlighted in blue.

Comment 4. As indicated at the end of the Abstract the value of this study is verylimited (3 spectra, no independent replicates, only one genotype). For aconclusion like “These findings emphasize the importance of optimizing LEDspectra to improve both productivity and nutritional quality” there is morethan enough proof without this study.

Response 4. We thank the reviewer for this valuable comment. We agree that studies on LED spectra have been widely conducted, particularly in mature basil plants, although studies on basil microgreens remain relatively limited. While LED 1 and LED 2 represent commonly used spectral combinations, LED 3 includes a more complex spectrum with far-red and white components, providing additional insight into the effects of extended spectral composition. In this context, the study offers a comparative evaluation of commonly used and modified light spectra under controlled vertical farming conditions.

Furthermore, as indicated above, the revised manuscript now clearly describes the experimental design, including independent replicates and sampling procedures, to address concerns regarding the robustness of the study. In addition, the conclusions have been revised to avoid overgeneralization and to better reflect the specific scope and contribution of the study.

Comment 5. Authors seem to copy-paste, otherwise how to explain line 569-570 “LED 3emerges as the most favorable treatment when prioritizing yield, biomass, andexternal fruit quality”. The study does not report on fruit, but on microgreensharvested 2 weeks after sowing.

Response 5. We thank the reviewer for this careful observation. We acknowledge that the use of the term “fruit quality” was inappropriate in the context of this study and likely resulted from an oversight during manuscript preparation. This has been corrected in the revised manuscript to accurately reflect the scope of the study, which focuses on basil microgreens. The relevant text has been revised and highlighted in blue.

**MıNOR COMMENTS**

Comment 1. Give treatments logic codes, not LED1, LED2 and LED3

Response 1. We thank the reviewer for this helpful suggestion. In order to maintain consistency and clarity throughout the manuscript, we have retained the treatment codes (LED 1, LED 2, and LED 3), which are consistently used in the text, tables, and figures. The meaning of these codes was already described in the Materials and Methods section and is also provided in the captions of the figures and tables. In addition, for further clarity, we have included an explicit definition of each treatment at its first occurrence in the Methods section (highlighted in blue). We believe this approach ensures both readability and interpretability without introducing inconsistencies across the manuscript.

Comment 2. Line 96: ‘most existing studies’ Provide references

Response 2. We thank the reviewer for this comment. In response, relevant references have been added to support the statement regarding “most existing studies.” Specifically, four recent studies have been incorporated into the revised manuscript to substantiate this claim. The revised text has been updated accordingly and highlighted in blue.

Comment 3. Line 110: How do you conclude that basil has a high yield potential?

Response 3. We thank the reviewer for this comment. The statement on high yield potential has been removed from the revised manuscript. The revised text is provided in the “Plant Material” section and highlighted in blue.

**RESPONSES TO THE COMMENTS PROVIDED BY REVIEWER 2 IN THE ATTACHED FILE**

Comment 1. The manuscript investigates the effects of different LED spectra on basil microgreens; however, similar studies are already abundant in the literature. The introduction does not sufficiently articulate the specific knowledge gap addressed by the present work. As a result, the novelty of the study appears moderate.

Response 1. We thank the reviewer for this valuable comment. In response, the Introduction has been revised to more clearly define the research gap and highlight the novelty of the study. These improvements have been incorporated into the last three paragraphs of the Introduction in the revised manuscript and are highlighted in blue.

Comment 2. The manuscript does not clearly specify the number of biological replicates, independent experimental repeats, or whether treatments were randomized. This lack of clarity raises concerns about the statistical robustness and reproducibility of the findings.

Response 2. We thank the reviewer for this comment. The Materials and Methods section has been revised to clearly define the experimental design, biological replicates, and randomization. The experiment was conducted as a completely randomized design with three independent biological replicates, each container considered as an experimental unit. The revised text is highlighted in blue.

Comment 3. While spectral quality is compared, it is not sufficiently clear whether photosynthetic photon flux density (PPFD) and daily light integral (DLI) were kept constant across treatments. Without proper normalization, observed effects may be due to differences in light intensity rather than spectral composition.

Response 3. We thank the reviewer for this important comment. In response, the Materials and Methods section has been revised to clearly indicate that PPFD was maintained constant across all treatments. In addition, the corresponding daily light integral (DLI) has been calculated and reported. The revised text is highlighted in blue.

Comment 4. The controlled-environment conditions (temperature stability, relative humidity, CO₂ concentration, airflow) are insufficiently described. Since microgreen growth is highly sensitive to microclimate variation, this omission may confound interpretation.

Response 4. We thank the reviewer for this important comment. In response, the Materials and Methods section has been revised to provide a more detailed description of the controlled-environment conditions, including temperature, relative humidity, CO₂ concentration, and airflow. These additions clarify that uniform microclimatic conditions were maintained across all treatments. The revised text is highlighted in blue.

Comment 5. The statistical treatment appears basic relative to the number of measured variables. There is no clear indication of correction for multiple comparisons, reporting of effect, sizes and multivariate analysis for nutritional parameters.

Response 5. We thank the reviewer for this valuable comment. The statistical approach used in this study includes analysis of variance (ANOVA) followed by the LSD test, which is a widely accepted method in horticultural research for comparing treatment means. In addition, multivariate analyses, including principal component analysis (PCA) and heat map visualization, were conducted to evaluate the relationships among the measured variables and to identify treatment-specific patterns. These analyses are presented in the manuscript and provide a comprehensive assessment of the dataset. Considering the scope and objectives of the study, we believe that the applied statistical methods are appropriate and sufficient to support the conclusions.

**The following points may consider as major comments minor comments:**

Comment 1. The manuscript contains several long and complex sentences that affect readability. Minor grammatical inconsistencies and redundancies are present.

Response 1. We thank the reviewer for this comment. The manuscript has been carefully revised to improve readability by simplifying long and complex sentences, correcting minor grammatical inconsistencies, and eliminating redundancies.

Comment 2. Maintain consistent use of units, abbreviations, and spectral terminology throughout the manuscript.

Response 2. We thank the reviewer for this helpful comment. The manuscript has been carefully revised to ensure consistent use of units, abbreviations, and spectral terminology throughout the text.

Comment 3. Consider incorporating a few more recent (last 2–3 years) references on LED optimization in microgreens to strengthen the literature positioning.

Response 3. We thank the reviewer for this suggestion. In response, recent references on LED optimization in microgreens have been incorporated into the manuscript, including one study from 2025 and three studies from 2026. The relevant references are listed below.

Da Young, L. E. E., Kim, M. J., In-Lee, C. H. O. I., Yongduk, K. I. M., Jidong, K. I. M., & Ho-Min, K. A. N. G. (2025). Effect of LED light quality on the growth and photomorphogenesis of basil in closed-type plant factory. *Notulae Botanicae Horti Agrobotanici Cluj-Napoca*, *53*(2), 14409-14409.

Bafrani, M. V. A., Mehrjerdi, M. Z., Aliniaeifard, S., & Seif, M. (2026). Optimizing LED light in controlled environments to enhance yield and nutritional quality of basil microgreens. *South African Journal of Botany*, *190*, 262-275.

Sobhanan, A., Meena, R. K., Dhaka, A. S., Mishra, G. P., & Nishad, J. (2026). Optimization of LED-Based Phototreatments for Enhancing Antioxidant Potential and Marketable Traits in Microgreens. *Applied Food Research*, 101918. SOBHANAN

Borrelli, A., Zamani, F., Logegaray, V.R. *et al.* Manipulation of light intensity and spectrum for driving growth and metabolism to improve nutritional and nutraceutical quality of microgreens. *Plant Growth Regul* **106**, 26 (2026). https://doi.org/10.1007/s10725-026-01434-7

Comment 4. **Overall Recommendation:Major revision** required before the manuscript can be considered for publication. The study is relevant and potentially valuable, but clarification of experimental rigor, statistical robustness and novelty positioning is needed.

Response 4. We thank the reviewer for this overall evaluation and constructive feedback. In response, the manuscript has been thoroughly revised to improve the clarity of the experimental design, strengthen the statistical analysis, and better highlight the novelty and scientific contribution of the study. All relevant revisions have been incorporated into the manuscript and are highlighted in blue.
