# Supplementary material for: Effects of LED spectral compositions on yield, growth, and nutritional quality of basil microgreens in indoor vertical farming
Source: PLoS One. 2026 Jul 7;21(7):e0352317. doi: 10.1371/journal.pone.0352317 (PMC13340805; doi:10.1371/journal.pone.0352317)
Supplement: Attachment — Submitted filename: Response to Reviewers (Reviewer 1) (1).docx [file pone.0352317.s001.docx]

**Response to Reviewer #1**

Dear Reviewer,
We sincerely thank you for your valuable and constructive feedback. Revisions have been highlighted in red in the revised manuscripts, and our point-by-point responses are provided below.

**Comment 1.** Line no. 18: L*, a*, b* - may be explained in foot note for clearity and easy understanding by the reader.

**Response 1:** In the revised manuscript, we have provided a clearer explanation of the L*, a*, and b* color parameters to facilitate better understanding for the readers.

**Comment 2.** Line no. 19: pH is a numerical scale that measures the acidity or alkalinity, so use of both needed explanation.

**Response 2:** To avoid confusion between pH and acidity, we clarified these terms in the Abstract with additional explanations in parentheses (pH: hydrogen ion concentration indicating whether a solution is acidic or alkaline; titratable acidity: the total amount of neutralizable acids).

**Comment 3.** In Introduction: The effect of spectral quality of light has been cited in may places which may be written clubbing all at one place.

**Response 3:** We have addressed the reviewer’s suggestion by consolidating all statements regarding the effects of light spectral quality in one section of the Introduction. Redundant mentions have been removed, and the relevant information is now presented in a clear and cohesive manner, while preserving all original references.

**Comment 4.** Line no. 90: What are the physiological and biochemical traits my be mentioned if available?

**Response 4:** In response to the comment on line 90, the section has been revised to provide a more detailed explanation of the measured parameters and their significance.

**Comment 5.** In Materials and Methods - Why only these three light spectra were used (LED 1, LED 2, and LED 3) a logical reasoning should be included? Is these are universally accepted spectra and no other combinations are possible.

**Response 5:** We appreciate the reviewer’s comment. Red and blue light combinations have been extensively studied in previous research; therefore, in our study, we designed LED 1 to represent a different ratio of these two wavelengths. In LED 3, far-red and white light were additionally incorporated alongside red and blue to explore their combined effects. LED 2 was included as a comparison using the universally accepted PAR range (400–700 nm). This design allowed us to evaluate both commonly used and modified light spectra and to provide a more comprehensive understanding of their influence on microgreen growth and quality.

**Comment 6.** Line No. 141: quantity/ percentage of P and K should be mentioned.

**Response 6:** We would like to inform you that, in addition to nitrogen (0.8%), the substrate used also contains approximately 0.24% phosphorus (P₂O₅) and 0.27% potassium (K₂O), which have been incorporated via the starter fertilizer.

**Comment 7.** Line No. 146: pure water - whether, filtered water, distilled water, and or tape water needs clearity. what is the mineral content in the water?

**Response 7:** We would like to clarify that the water used in this study was obtained through a reverse osmosis system. This process effectively removes nearly all minerals and impurities, so the water can be considered virtually free of dissolved minerals.

**Comment 8.** Line No. 152: Number of time of irrigation/ day/ week include the volume of nutrient solution used for irrigation in each of the microgreen selves.

**Response 8:** The necessary clarification has been incorporated into the revised manuscript.

**Comment 9.** Line No. 155: Method of measuring the growth rate unexplained before harvest./ the sentance need rewriting if no measurement has been done.

**Response 9:**The Plant Harvest section has been revised to provide a clearer and more detailed description of the harvesting criteria.

**Comment 10.** Line No. 166: Formula for Yield per unit area is missing.

**Response 10:** Yield per unit as g/cm^2^ was edited.

**Comment 11.** Line No. 186: Asidity -replace with acidity.

**Response 11:** Thank you for pointing this out—it has now been corrected to “acidity.”

**Comment 12.** Table No. 1,2,3,4: a, b, c - need to be explained in footnote for clearity.

**Response 12:** In response, we have added an explanation under the tables and figures.

**Comment 13.** Line No 417: color a - same alphabet 'a' is used to represent coloum as well colors creating confusion - if possible they may use unique alphabet.

**Response 13:** We would like to clarify that the letter “a” used here refers to color analysis, not statistical grouping. This notation is a standard method commonly used in the literature to describe spectral components (e.g., red, blue, far-red). Therefore, although the same letter appears in both contexts, their meanings are distinct: one indicates the color treatment, while the other represents statistical differences. We hope this clarification addresses your concern.

**Comment 14.** Line No 552: the statement In contrast,................by light spectrum alone may be revised. - In contrast..... also influenced by other factors such as ............... light spectrum alone.

**Response 14:** We have revised the sentence to clarify the meaning, ensuring that the influence of both light spectrum and other factors is clearly distinguished.

**Comment 15.** Line No 613: Future studies....... / may be revised.

**Response 15:** The sentence regarding future studies has been revised to make it clearer and more specific, emphasizing the interactions between light spectra, cultivar traits, and environmental factors for refining lighting strategies in commercial microgreen cultivation.

**Comment 16.** There is a scope for further improvement in presenting the findings.

**Response 16:** In response, we have revised the presentation of the findings to improve clarity and readability, including adjustments in the tables, figures, and textual descriptions where necessary.
