# Supplementary material for: Effects of LED spectral compositions on yield, growth, and nutritional quality of basil microgreens in indoor vertical farming
Source: PLoS One. 2026 Jul 7;21(7):e0352317. doi: 10.1371/journal.pone.0352317 (PMC13340805; doi:10.1371/journal.pone.0352317)
Supplement: Attachment — Submitted filename: Comment.docx [file pone.0352317.s002.docx]

**Comments for the article PONE-D-25-35263R1**

The study evaluates different LED spectra on basil microgreens — a topic that is already heavily studied. Hence, the paper lacks the novelty of the work.

The following points may consider as **major comments:**

1. The manuscript investigates the effects of different LED spectra on basil microgreens; however, similar studies are already abundant in the literature. The introduction does not sufficiently articulate the specific knowledge gap addressed by the present work. As a result, the novelty of the study appears moderate.
2. The manuscript does not clearly specify the number of biological replicates, independent experimental repeats, or whether treatments were randomized. This lack of clarity raises concerns about the statistical robustness and reproducibility of the findings.
3. While spectral quality is compared, it is not sufficiently clear whether photosynthetic photon flux density (PPFD) and daily light integral (DLI) were kept constant across treatments. Without proper normalization, observed effects may be due to differences in light intensity rather than spectral composition.
4. The controlled-environment conditions (temperature stability, relative humidity, CO₂ concentration, airflow) are insufficiently described. Since microgreen growth is highly sensitive to microclimate variation, this omission may confound interpretation.
5. The statistical treatment appears basic relative to the number of measured variables. There is no clear indication of correction for multiple comparisons, reporting of effect, sizes and multivariate analysis for nutritional parameters

**The following points may consider as major comments Minor Comments:**

1. The manuscript contains several long and complex sentences that affect readability. Minor grammatical inconsistencies and redundancies are present.
2. Maintain consistent use of units, abbreviations, and spectral terminology throughout the manuscript.
3. Consider incorporating a few more recent (last 2–3 years) references on LED optimization in microgreens to strengthen the literature positioning.

**Overall Recommendation:**

**Major revision** required before the manuscript can be considered for publication. The study is relevant and potentially valuable, but clarification of experimental rigor, statistical robustness and novelty positioning is needed.
