# Supplementary material for: Effects of LED spectral compositions on yield, growth, and nutritional quality of basil microgreens in indoor vertical farming
Source: PLoS One. 2026 Jul 7;21(7):e0352317. doi: 10.1371/journal.pone.0352317 (PMC13340805; doi:10.1371/journal.pone.0352317)
Supplement: Attachment — Submitted filename: Response to editor comment May 22, 2026.docx [file pone.0352317.s004.docx]

May 15, 2026

Dear Editor Dr. Šiler,

Thank you very much for your valuable comment and careful evaluation of our manuscript. We sincerely appreciate your observation regarding the visual appearance of the error bars in Figure 5. Following your recommendation, we carefully re-examined both the statistical outputs and the graphical presentation of the data. The issue you highlighted has been addressed in detail below, and the figure has been revised accordingly.

In Figure 5, the error bars in the Color L* and Color b* graphs appeared visually large. Upon re-examining the data, we confirmed that the standard error values were not actually high and that the replicates were relatively close to each other. However, we noticed that the visual appearance of the error bars was mainly caused by the scaling of the y-axis during graph preparation.

In the previous version (Old Version of Figure 5), the y-axis values were displayed at very narrow intervals. For example, in the Color L* graph, the scale was arranged as 86, 86.5, 87, 87.5, etc., while in the Color b* graph it was arranged as 0, 0.5, 1.0, 1.5, 2.0, etc. Such narrow intervals visually exaggerated the error bars.

Therefore, the graphs have now been revised (New Version Figure 5). In the updated Color L* graph, the y-axis values are presented at intervals of 5 units (50, 55, 60, 65, 70, 75, etc.). Similarly, in the Color a* and Color b* graphs, the y-axis values are now presented at intervals of 1 unit (0, 1, 2, 3, 4, 5, 6, etc.). In this revised form, the standard error bars no longer appear artificially inflated as in the previous version.

Since there were no changes in the original data or statistical outputs related to the color parameters (L*, a*, and b*), no revisions were required in the Results or Discussion sections of the manuscript. Accordingly, only Figure 5 was revised in the current submission.

We respectfully submit these revisions for your consideration and kindly hope that the manuscript can now be accepted for publication in its final revised form.

Sincerely,

Prof. Dr. H. Yildiz Dasgan

Corresponding author

Old Version of Figure 5.

New Version of Figure 5.
