# Supplementary material for: Effects of LED spectral compositions on yield, growth, and nutritional quality of basil microgreens in indoor vertical farming
Source: PLoS One. 2026 Jul 7;21(7):e0352317. doi: 10.1371/journal.pone.0352317 (PMC13340805; doi:10.1371/journal.pone.0352317)
Supplement: Attachment — Submitted filename: RESPONSE TO EDITORIAL COMMENTS.docx [file pone.0352317.s005.docx]

**RESPONSE TO EDITORIAL COMMENTS**

Dear Editor,

Thank you for your comments and for giving us the opportunity to improve our manuscript. We have carefully addressed the issues raised and made the necessary revisions. Our responses are provided below.

Comment 1:
Please improve the quality/resolution of Figure 5.

Response:
The resolution of Figure 5 has been increased to improve image quality and readability. The revised figure has been uploaded and incorporated into the revised manuscript.

Comment 2:
Please correct the unclear lines in Figure 6.

Response:
The unclear lines in Figure 6 have been corrected and the figure has been revised to improve clarity and visual presentation. The updated figure has been incorporated into the revised manuscript.

All revised figures and the updated manuscript have been uploaded to the submission system.

Thank you for your consideration.

Sincerely,

Prof. Dr. H. Yıldız Daşgan
Corresponding Author

Bu format editörlerin istediği klasik "Comment–Response" (point-by-point response) formatına tam uygundur.
