# Supplementary material for: Effects of LED spectral compositions on yield, growth, and nutritional quality of basil microgreens in indoor vertical farming
Source: PLoS One. 2026 Jul 7;21(7):e0352317. doi: 10.1371/journal.pone.0352317 (PMC13340805; doi:10.1371/journal.pone.0352317)
Supplement: Peer review history

# Peer review: Decision Letter 0

**DOI:** 10.1371/journal.pone.0352317.r001

**Authors:** Partha Kalita

1 Sep 2025

PONE-D-25-35263

Effects of LED Spectral Compositions on Yield, Growth, and Nutritional Quality of Basil Microgreens in Indoor Vertical Farming

PLOS ONE

Dear Dr. Dasgan,

Thank you for submitting your manuscript to PLOS ONE. After careful consideration, we feel that it has merit but does not fully meet PLOS ONE’s publication criteria as it currently stands. Therefore, we invite you to submit a revised version of the manuscript that addresses the points raised during the review process.

==============================

**ACADEMIC EDITOR:**

Please correct according to the reviewer's comments and resubmit.

==============================

Please submit your revised manuscript by Oct 16 2025 11:59PM. If you will need more time than this to complete your revisions, please reply to this message or contact the journal office at plosone@plos.org. When you're ready to submit your revision, log on to https://www.editorialmanager.com/pone/ and select the 'Submissions Needing Revision' folder to locate your manuscript file.

Please include the following items when submitting your revised manuscript:

- A rebuttal letter that responds to each point raised by the academic editor and reviewer(s). You should upload this letter as a separate file labeled 'Response to Reviewers'.
- A marked-up copy of your manuscript that highlights changes made to the original version. You should upload this as a separate file labeled 'Revised Manuscript with Track Changes'.
- An unmarked version of your revised paper without tracked changes. You should upload this as a separate file labeled 'Manuscript'.

If you would like to make changes to your financial disclosure, please include your updated statement in your cover letter. Guidelines for resubmitting your figure files are available below the reviewer comments at the end of this letter.

If applicable, we recommend that you deposit your laboratory protocols in protocols.io to enhance the reproducibility of your results. Protocols.io assigns your protocol its own identifier (DOI) so that it can be cited independently in the future. For instructions see: https://journals.plos.org/plosone/s/submission-guidelines#loc-laboratory-protocols. Additionally, PLOS ONE offers an option for publishing peer-reviewed Lab Protocol articles, which describe protocols hosted on protocols.io. Read more information on sharing protocols at https://plos.org/protocols?utm_medium=editorial-email&utm_source=authorletters&utm_campaign=protocols.

We look forward to receiving your revised manuscript.

Kind regards,

Partha Pratim Kalita, PhD

Academic Editor

PLOS ONE

Journal Requirements:

When submitting your revision, we need you to address these additional requirements.

1. Please ensure that your manuscript meets PLOS ONE's style requirements, including those for file naming. The PLOS ONE style templates can be found at

https://journals.plos.org/plosone/s/file?id=wjVg/PLOSOne_formatting_sample_main_body.pdf and

https://journals.plos.org/plosone/s/file?id=ba62/PLOSOne_formatting_sample_title_authors_affiliations.pdf.

2. Please include a complete copy of PLOS’ questionnaire on inclusivity in global research in your revised manuscript. Our policy for research in this area aims to improve transparency in the reporting of research performed outside of researchers’ own country or community. The policy applies to researchers who have travelled to a different country to conduct research, research with Indigenous populations or their lands, and research on cultural artefacts. The questionnaire can also be requested at the journal’s discretion for any other submissions, even if these conditions are not met.  Please find more information on the policy and a link to download a blank copy of the questionnaire here: https://journals.plos.org/plosone/s/best-practices-in-research-reporting. Please upload a completed version of your questionnaire as Supporting Information when you resubmit your manuscript.

3.  We note that the grant information you provided in the ‘Funding Information’ and ‘Financial Disclosure’ sections do not match.

When you resubmit, please ensure that you provide the correct grant numbers for the awards you received for your study in the ‘Funding Information’ section.

4.  Thank you for stating the following financial disclosure:

[Research Foundation Office of Cukurova University (BAP), Project No. FBA-2024-15159].

Please state what role the funders took in the study.  If the funders had no role, please state: ""The funders had no role in study design, data collection and analysis, decision to publish, or preparation of the manuscript.""

If this statement is not correct you must amend it as needed.

Please include this amended Role of Funder statement in your cover letter; we will change the online submission form on your behalf.

5. If the reviewer comments include a recommendation to cite specific previously published works, please review and evaluate these publications to determine whether they are relevant and should be cited. There is no requirement to cite these works unless the editor has indicated otherwise.

Additional Editor Comments:

Reviewer #1:

Minor Revision

Reviewer #2:

Major Revision

[Note: HTML markup is below. Please do not edit.]

Reviewers' comments:

Reviewer's Responses to Questions

**Comments to the Author**

1. Is the manuscript technically sound, and do the data support the conclusions?

The manuscript must describe a technically sound piece of scientific research with data that supports the conclusions. Experiments must have been conducted rigorously, with appropriate controls, replication, and sample sizes. The conclusions must be drawn appropriately based on the data presented.

Reviewer #1: Yes

Reviewer #2: Partly

**********

2. Has the statistical analysis been performed appropriately and rigorously?

Reviewer #1: Yes

Reviewer #2: Yes

**********

3. Have the authors made all data underlying the findings in their manuscript fully available?

The PLOS Data policy requires authors to make all data underlying the findings described in their manuscript fully available without restriction, with rare exception (please refer to the Data Availability Statement in the manuscript PDF file). The data should be provided as part of the manuscript or its supporting information, or deposited to a public repository. For example, in addition to summary statistics, the data points behind means, medians and variance measures should be available. If there are restrictions on publicly sharing data—e.g. participant privacy or use of data from a third party—those must be specified.

Reviewer #1: Yes

Reviewer #2: No

**********

4. Is the manuscript presented in an intelligible fashion and written in standard English?

PLOS ONE does not copyedit accepted manuscripts, so the language in submitted articles must be clear, correct, and unambiguous. Any typographical or grammatical errors should be corrected at revision, so please note any specific errors here.

Reviewer #1: Yes

Reviewer #2: No

**********

5. Review Comments to the Author

Please use the space provided to explain your answers to the questions above. You may also include additional comments for the author, including concerns about dual publication, research ethics, or publication ethics. (Please upload your review as an attachment if it exceeds 20,000 characters)

Reviewer #1: I have gone thorough the article "Effects of LED Spectral Compositions on Yield, Growth and Nutritional Quality of Basil Microgreens in Indoor Vertical Farming". The authors in this study investigated the effect of three different combination of light spectrum on the growth, yield and nutritional quality and yield of the basil microgreens and presented their findings with appropriate statistical measurements. However, there are few points needed clarification and improvements.

1. Line no. 18: L*, a*, b* - may be explained in foot note for clearity and easy understanding by the reader.

2. Line no. 19: pH is a numerical scale that measures the acidity or alkalinity, so use of both needed explanation.

3. In Introduction: The effect of spectral quality of light has been cited in may places which may be written clubbing all at one place.

4. Line no. 90: What are the physiological and biochemical traits my be mentioned if available?

5. In Materials and Methods - Why only these three light spectra were used (LED 1, LED 2, and LED 3) a logical reasoning should be included? Is these are universally accepted spectra and no other combinations are possible.

6. Line No. 141: quantity/ percentage of P and K should be mentioned.

7. Line No. 146: pure water - whether, filtered water, distilled water, and or tape water needs clearity. what is the mineral content in the water?

8. Line No. 152: Number of time of irrigation/ day/ week include the volume of nutrient solution used for irrigation in each of the microgreen selves.

9. Line No. 155: Method of measuring the growth rate unexplained before harvest./ the sentance need rewriting if no measurement has been done.

10. Line No. 166: Formula for Yield per unit area is missing.

11. Line No. 186: Asidity -replace with acidity

12. Table No. 1,2,3,4: a, b, c - need to be explained in footnote for clearity

13. Line No 417: color a - same alphabet 'a' is used to represent coloum as well colors creating confusion - if possible they may use unique alphabet.

14. Line No 552: the statement In contrast,................by light spectrum alone may be revised. - In contrast..... also influenced by other factors such as ............... light spectrum alone.

15. Line No 613: Future studies....... / may be revised.

16. There is a scope for further improvement in presenting the findings.

Reviewer #2: Major Revision – The study is strong in scope and data collection, but revisions are necessary to improve clarity, highlight novelty, balance the discussion, and ensure data transparency. Addressing the above points will significantly enhance the manuscript’s impact and readability.

**********

6. PLOS authors have the option to publish the peer review history of their article (what does this mean?). If published, this will include your full peer review and any attached files.

If you choose “no”, your identity will remain anonymous but your review may still be made public.

**Do you want your identity to be public for this peer review?**  For information about this choice, including consent withdrawal, please see our Privacy Policy.

Reviewer #1: No

Reviewer #2: No

**********

[NOTE: If reviewer comments were submitted as an attachment file, they will be attached to this email and accessible via the submission site. Please log into your account, locate the manuscript record, and check for the action link "View Attachments". If this link does not appear, there are no attachment files.]

While revising your submission, please upload your figure files to the Preflight Analysis and Conversion Engine (PACE) digital diagnostic tool, https://pacev2.apexcovantage.com/. PACE helps ensure that figures meet PLOS requirements. To use PACE, you must first register as a user. Registration is free. Then, login and navigate to the UPLOAD tab, where you will find detailed instructions on how to use the tool. If you encounter any issues or have any questions when using PACE, please email PLOS at figures@plos.org. Please note that Supporting Information files do not need this step.

# Author comment: Author response to Decision Letter 1

**DOI:** 10.1371/journal.pone.0352317.r002

12 Oct 2025

Point-by-point responses to reviewer comments have been prepared and uploaded as a separate file in the system

Attachment Submitted filename: Response to Reviewers (Reviewer 1) (1).docx

# Peer review: Decision Letter 1

**DOI:** 10.1371/journal.pone.0352317.r003

**Authors:** Partha Kalita, Branislav Šiler

2 Mar 2026

PONE-D-25-35263R1

Effects of LED Spectral Compositions on Yield, Growth, and Nutritional Quality of Basil Microgreens in Indoor Vertical Farming

PLOS One

Dear Dr. Dasgan,

Thank you for submitting your manuscript to PLOS ONE. After careful consideration, we feel that it has merit but does not fully meet PLOS ONE’s publication criteria as it currently stands. Therefore, we invite you to submit a revised version of the manuscript that addresses the points raised during the review process.

==============================

**The authors are advised to put additional efforts in meticulously addressing several major omissions listed in the reviewers' comments. Reviewer #2 and Reviewer #3 encouraged authors to more clearly articulate the specific research gap this study fills and to address the mechanistic hypothesis, i.e., what are the expected advances of red/blues light over the full light spectrum as well as possible advances of involvement/skipping the far-red wavelengths. Moreover, Reviewer #2 suggested to explain how the experimental design or findings differ meaningfully from prior studies, and the unique practical or scientific contribution of the work.**

**Statistical testing information is missing and the existence of experimental replications is questionable.**

**Finally, the authors are advised not to overestimate their findings when presenting conclusions of the study.**

**Using the expressions such as "fruit quality" and similar implies too generative content.**

**Please mind the attachment containing additional comments provided by Reviewer #2.**

==============================

Please submit your revised manuscript by Apr 16 2026 11:59PM. If you will need more time than this to complete your revisions, please reply to this message or contact the journal office at plosone@plos.org. When you're ready to submit your revision, log on to https://www.editorialmanager.com/pone/ and select the 'Submissions Needing Revision' folder to locate your manuscript file.

Please include the following items when submitting your revised manuscript:

- A letter that responds to each point raised by the academic editor and reviewer(s). You should upload this letter as a separate file labeled 'Response to Reviewers'.
- A marked-up copy of your manuscript that highlights changes made to the original version. You should upload this as a separate file labeled 'Revised Manuscript with Track Changes'.
- An unmarked version of your revised paper without tracked changes. You should upload this as a separate file labeled 'Manuscript'.

If you would like to make changes to your financial disclosure, please include your updated statement in your cover letter. Guidelines for resubmitting your figure files are available below the reviewer comments at the end of this letter.

If applicable, we recommend that you deposit your laboratory protocols in protocols.io to enhance the reproducibility of your results. Protocols.io assigns your protocol its own identifier (DOI) so that it can be cited independently in the future. For instructions see: https://journals.plos.org/plosone/s/submission-guidelines#loc-laboratory-protocols. Additionally, PLOS ONE offers an option for publishing peer-reviewed Lab Protocol articles, which describe protocols hosted on protocols.io. Read more information on sharing protocols at https://plos.org/protocols?utm_medium=editorial-email&utm_source=authorletters&utm_campaign=protocols.

We look forward to receiving your revised manuscript.

Kind regards,

Branislav T. Šiler, Ph.D.

Academic Editor

PLOS One

Journal Requirements:

If the reviewer comments include a recommendation to cite specific previously published works, please review and evaluate these publications to determine whether they are relevant and should be cited. There is no requirement to cite these works unless the editor has indicated otherwise.

[Note: HTML markup is below. Please do not edit.]

Reviewers' comments:

Reviewer's Responses to Questions

**Comments to the Author**

1. If the authors have adequately addressed your comments raised in a previous round of review and you feel that this manuscript is now acceptable for publication, you may indicate that here to bypass the “Comments to the Author” section, enter your conflict of interest statement in the “Confidential to Editor” section, and submit your "Accept" recommendation.

Reviewer #1: All comments have been addressed

Reviewer #2: All comments have been addressed

Reviewer #3: (No Response)

**********

2. Is the manuscript technically sound, and do the data support the conclusions?

The manuscript must describe a technically sound piece of scientific research with data that supports the conclusions. Experiments must have been conducted rigorously, with appropriate controls, replication, and sample sizes. The conclusions must be drawn appropriately based on the data presented.

Reviewer #1: Yes

Reviewer #2: Partly

Reviewer #3: No

**********

3. Has the statistical analysis been performed appropriately and rigorously?

Reviewer #1: Yes

Reviewer #2: No

Reviewer #3: No

**********

4. Have the authors made all data underlying the findings in their manuscript fully available?

The PLOS Data policy requires authors to make all data underlying the findings described in their manuscript fully available without restriction, with rare exception (please refer to the Data Availability Statement in the manuscript PDF file). The data should be provided as part of the manuscript or its supporting information, or deposited to a public repository. For example, in addition to summary statistics, the data points behind means, medians and variance measures should be available. If there are restrictions on publicly sharing data—e.g. participant privacy or use of data from a third party—those must be specified.

Reviewer #1: Yes

Reviewer #2: Yes

Reviewer #3: No

**********

5. Is the manuscript presented in an intelligible fashion and written in standard English?

PLOS ONE does not copyedit accepted manuscripts, so the language in submitted articles must be clear, correct, and unambiguous. Any typographical or grammatical errors should be corrected at revision, so please note any specific errors here.

Reviewer #1: Yes

Reviewer #2: No

Reviewer #3: No

**********

6. Review Comments to the Author

Please use the space provided to explain your answers to the questions above. You may also include additional comments for the author, including concerns about dual publication, research ethics, or publication ethics. (Please upload your review as an attachment if it exceeds 20,000 characters)

Reviewer #1: The authors have adequately addressed the comments raised in a previous round of review and you feel that this manuscript is now acceptable for publication.

Reviewer #2: Major revision required before the manuscript can be considered for publication. The study is relevant and potentially valuable, but clarification of experimental rigor, statistical robustness and novelty positioning is needed.

Reviewer #3: This manuscript describes the effects of 3 light spectra on growth and quality parameters of microgreen basil in a 2 week experiment.

The manuscript has serious flaws:

1. It is mentioned that ANOVA is used, however, statistical information is missing. Manuscript gives the impression that there have been no independent repetitions. Line 112: 3 layers each illuminated by a different light spectrum. It could easily occur that the top layer has a somewhat higher or lower temperature, and without proper randomization and repetition a temperature effect would be wrongly seen as a light spectrum effect.

2. Only 3 light spectra are compared but there is no logic provided, and the figures of the spectra are not given (a full spectrum within 400-700 nm is not enough information).

3. There is no mechanistic hypothesis, e.g. what would be expected from only R:B versus full spectrum and why? Or from a full spectrum with or without FR and why? The hypothesis given is merely: we expect effects of LED spectrum on the variables we measure.

4. As indicated at the end of the Abstract the value of this study is very limited (3 spectra, no independent replicates, only one genotype). For a conclusion like “These findings emphasize the importance of optimizing LED spectra to improve both productivity and nutritional quality” there is more than enough proof without this study.

5. Authors seem to copy-paste, otherwise how to explain line 569-570 “LED 3 emerges as the most favorable treatment when prioritizing yield, biomass, and external fruit quality”. The study does not report on fruit, but on microgreens harvested 2 weeks after sowing.

Minor comments

Give treatments logic codes, not LED1, LED2 and LED3

Line 96: ‘most existing studies’ Provide references

Line 110: How do you conclude that basil has a high yield potential?

**********

7. PLOS authors have the option to publish the peer review history of their article (what does this mean?). If published, this will include your full peer review and any attached files.

If you choose “no”, your identity will remain anonymous but your review may still be made public.

**Do you want your identity to be public for this peer review?**  For information about this choice, including consent withdrawal, please see our Privacy Policy.

Reviewer #1: No

Reviewer #2: No

Reviewer #3: No

**********

[NOTE: If reviewer comments were submitted as an attachment file, they will be attached to this email and accessible via the submission site. Please log into your account, locate the manuscript record, and check for the action link "View Attachments". If this link does not appear, there are no attachment files.]

To ensure your figures meet our technical requirements, please review our figure guidelines: https://journals.plos.org/plosone/s/figures

You may also use PLOS’s free figure tool, NAAS, to help you prepare publication quality figures: https://journals.plos.org/plosone/s/figures#loc-tools-for-figure-preparation.

NAAS will assess whether your figures meet our technical requirements by comparing each figure against our figure specifications.

Attachment Submitted filename: Comment.docx

# Author comment: Author response to Decision Letter 2

**DOI:** 10.1371/journal.pone.0352317.r004

7 Apr 2026

Dear Editor and Reviewers,

We would like to express our sincere gratitude for your constructive comments, and insightful suggestions, which have significantly contributed to improving the quality of our manuscript. All comments have been carefully considered and thoroughly addressed. We have provided detailed, point-by-point responses to each comment below. In addition, all revisions in the manuscript have been clearly indicated using track changes and highlighted in blue for ease of evaluation.

In accordance with the journal’s request, we have included a complete copy of the “Inclusivity in Global Research” questionnaire and uploaded it as a separate Supporting Information file during resubmission. In addition, all figures have been removed from the main manuscript file, and each figure has been uploaded separately to the system in TIFF format, as required.

EDITOR COMMENTS

Comment 1. The authors are advised to put additional efforts in meticulously addressing several major omissions listed in the reviewers' comments. Reviewer #2 and Reviewer #3 encouraged authors to more clearly articulate the specific research gap this study fills and to address the mechanistic hypothesis, i.e., what arethe expected advances of red/blues light over the full light spectrum as well as possible advances of involvement/skipping the far-red wavelengths. Moreover, Reviewer #2 suggested to explain how the experimental design or findings differ meaningfully from prior studies, and the unique practical or scientific contribution of the work.

Response 1. We thank the reviewers for these valuable and constructive suggestions. In response, we have revised the Introduction section to better clarify the research gap, underlying mechanistic hypothesis, and the expected advantages of different light spectra, including the role of far-red wavelengths. In addition, the novelty and the practical and scientific contributions of the study have been more clearly articulated. These revisions have been incorporated in the final three paragraphs of the Introduction (highlighted in blue).

Comment 2. Statistical testing information is missing and the existence of experimental replications is questionable.

Response 2: In response to the editor’s comment, the following information has been added to the Materials and Methods section and is highlighted in blue: “The experiment was conducted with three replicates, each replicate consisting of three containers. A total of 9 containers were placed on the multilayer shelves under each LED light spectrum. Each container contained approximately 3,500 microgreen basil plants. The experiment was arranged in a completely randomized design. Measurements and analyses were performed using plant samples collected from independent replicates. For yield calculations (g m⁻²), the total fresh weight of all microgreen plants harvested from each container was treated as a single replicate.”

Comment 3. Finally, the authors are advised not to overestimate their findings whenpresenting conclusions of the study. Using the expressions such as "fruit quality" and similar implies toogenerative content.

Response 3: We thank the reviewer for this valuable comment. We have carefully considered this point and revised the manuscript accordingly by moderating our statements and avoiding overly general expressions such as ‘fruit quality,’ ensuring that the conclusions are presented in a more precise and scientifically accurate manner.

RESPONSES TO REVIEWER 3 COMMENTS

Reviewer #3: This manuscript describes the effects of 3 light spectra on growthand quality parameters of microgreen basil in a 2 week experiment. The manuscript has serious flaws:

Comment 1. It is mentioned that ANOVA is used, however, statistical information ismissing. Manuscript gives the impression that there have been no independentrepetitions. Line 112: 3 layers each illuminated by a different light spectrum.It could easily occur that the top layer has a somewhat higher or lowertemperature, and without proper randomization and repetition a temperatureeffect would be wrongly seen as a light spectrum effect.

Response 1. We thank the reviewer for this valuable comment. We have revised the Materials and Methods section to clearly describe the experimental design and replication. The experiment was conducted in a completely randomized design with three independent replicates, and each container was defined as an experimental unit. Relevant details regarding replication and sampling procedures have been added and highlighted in blue. The statistical analysis, including ANOVA and LSD test (p < 0.05), was already described in the manuscript. These revisions aim to clarify the independence of replicates and the statistical approach used.

We thank the reviewer for this important observation. The vertical farming system used in this study was operated under controlled environmental conditions, with a well-established air circulation and climate control system ensuring uniform temperature distribution across all layers. Continuous air movement and regulated ventilation minimized the possibility of temperature gradients between the upper and lower shelves. Therefore, potential temperature differences among layers were considered negligible. In addition, the experimental design and replication structure have been clearly described in the revised manuscript to ensure that the observed effects can be attributed to the light spectrum treatments rather than environmental variation.

Comment 2. Only 3 light spectra are compared but there is no logic provided, and thefigures of the spectra are not given (a full spectrum within 400-700 nm is notenough information).

Response 2. We thank the reviewer for this valuable comment. In response, the spectral distribution graphs of each LED treatment have been added to the revised manuscript as Figure 2. These figures provide a clear representation of the light spectra based on the manufacturer-provided peak wavelengths and relative proportions of the spectral components. The relevant additions have been incorporated into the manuscript and highlighted in blue.

Comment 3. There is no mechanistic hypothesis, e.g. what would be expected from onlyR:B versus full spectrum and why? Or from a full spectrum with or without FRand why? The hypothesis given is merely: we expect effects of LED spectrum onthe variables we measure.

Response 3. We thank the reviewer for this insightful comment. We agree that the initial version of the manuscript did not sufficiently present the mechanistic basis of the hypothesis. In response, the Introduction section has been revised to clearly describe the expected physiological and photomorphogenic effects of red–blue light, full-spectrum light, and far-red inclusion. The revised text now explains the underlying mechanisms and the rationale for comparing these light treatments. These additions have been incorporated into the last three paragraphs of the Introduction in the revised manuscript and are highlighted in blue.

Comment 4. As indicated at the end of the Abstract the value of this study is verylimited (3 spectra, no independent replicates, only one genotype). For aconclusion like “These findings emphasize the importance of optimizing LEDspectra to improve both productivity and nutritional quality” there is morethan enough proof without this study.

Response 4. We thank the reviewer for this valuable comment. We agree that studies on LED spectra have been widely conducted, particularly in mature basil plants, although studies on basil microgreens remain relatively limited. While LED 1 and LED 2 represent commonly used spectral combinations, LED 3 includes a more complex spectrum with far-red and white components, providing additional insight into the effects of extended spectral composition. In this context, the study offers a comparative evaluation of commonly used and modified light spectra under controlled vertical farming conditions.

Furthermore, as indicated above, the revised manuscript now clearly describes the experimental design, including independent replicates and sampling procedures, to address concerns regarding the robustness of the study. In addition, the conclusions have been revised to avoid overgeneralization and to better reflect the specific scope and contribution of the study.

Comment 5. Authors seem to copy-paste, otherwise how to explain line 569-570 “LED 3emerges as the most favorable treatment when prioritizing yield, biomass, andexternal fruit quality”. The study does not report on fruit, but on microgreensharvested 2 weeks after sowing.

Response 5. We thank the reviewer for this careful observation. We acknowledge that the use of the term “fruit quality” was inappropriate in the context of this study and likely resulted from an oversight during manuscript preparation. This has been corrected in the revised manuscript to accurately reflect the scope of the study, which focuses on basil microgreens. The relevant text has been revised and highlighted in blue.

MıNOR COMMENTS

Comment 1. Give treatments logic codes, not LED1, LED2 and LED3

Response 1. We thank the reviewer for this helpful suggestion. In order to maintain consistency and clarity throughout the manuscript, we have retained the treatment codes (LED 1, LED 2, and LED 3), which are consistently used in the text, tables, and figures. The meaning of these codes was already described in the Materials and Methods section and is also provided in the captions of the figures and tables. In addition, for further clarity, we have included an explicit definition of each treatment at its first occurrence in the Methods section (highlighted in blue). We believe this approach ensures both readability and interpretability without introducing inconsistencies across the manuscript.

Comment 2. Line 96: ‘most existing studies’ Provide references

Response 2. We thank the reviewer for this comment. In response, relevant references have been added to support the statement regarding “most existing studies.” Specifically, four recent studies have been incorporated into the revised manuscript to substantiate this claim. The revised text has been updated accordingly and highlighted in blue.

Comment 3. Line 110: How do you conclude that basil has a high yield potential?

Response 3. We thank the reviewer for this comment. The statement on high yield potential has been removed from the revised manuscript. The revised text is provided in the “Plant Material” section and highlighted in blue.

RESPONSES TO THE COMMENTS PROVIDED BY REVIEWER 2 IN THE ATTACHED FILE

Comment 1. The manuscript investigates the effects of different LED spectra on basil microgreens; however, similar studies are already abundant in the literature. The introduction does not sufficiently articulate the specific knowledge gap addressed by the present work. As a result, the novelty of the study appears moderate.

Response 1. We thank the reviewer for this valuable comment. In response, the Introduction has been revised to more clearly define the research gap and highlight the novelty of the study. These improvements have been incorporated into the last three paragraphs of the Introduction in the revised manuscript and are highlighted in blue.

Comment 2. The manuscript does not clearly specify the number of biological replicates, independent experimental repeats, or whether treatments were randomized. This lack of clarity raises concerns about the statistical robustness and reproducibility of the findings.

Response 2. We thank the reviewer for this comment. The Materials and Methods section has been revised to clearly define the experimental design, biological replicates, and randomization. The experiment was conducted as a completely randomized design with three independent biological replicates, each container considered as an experimental unit. The revised text is highlighted in blue.

Comment 3. While spectral quality is compared, it is not sufficiently clear whether photosynthetic photon flux density (PPFD) and daily light integral (DLI) were kept constant across treatments. Without proper normalization, observed effects may be due to differences in light intensity rather than spectral composition.

Response 3. We thank the reviewer for this important comment. In response, the Materials and Methods section has been revised to clearly indicate that PPFD was maintained constant across all treatments. In addition, the corresponding daily light integral (DLI) has been calculated and reported. The revised text is highlighted in blue.

Comment 4. The controlled-environment conditions (temperature stability, relative humidity, CO₂ concentration, airflow) are insufficiently described. Since microgreen growth is highly sensitive to microclimate variation, this omission may confound interpretation.

Response 4. We thank the reviewer for this important comment. In response, the Materials and Methods section has been revised to provide a more detailed description of the controlled-environment conditions, including temperature, relative humidity, CO₂ concentration, and airflow. These additions clarify that uniform microclimatic conditions were maintained across all treatments. The revised text is highlighted in blue.

Comment 5. The statistical treatment appears basic relative to the number of measured variables. There is no clear indication of correction for multiple comparisons, reporting of effect, sizes and multivariate analysis for nutritional parameters.

Response 5. We thank the reviewer for this valuable comment. The statistical approach used in this study includes analysis of variance (ANOVA) followed by the LSD test, which is a widely accepted method in horticultural research for comparing treatment means. In addition, multivariate analyses, including principal component analysis (PCA) and heat map visualization, were conducted to evaluate the relationships among the measured variables and to identify treatment-specific patterns. These analyses are presented in the manuscript and provide a comprehensive assessment of the dataset. Considering the scope and objectives of the study, we believe that the applied statistical methods are appropriate and sufficient to support the conclusions.

The following points may consider as major comments minor comments:

Comment 1. The manuscript contains several long and complex sentences that affect readability. Minor grammatical inconsistencies and redundancies are present.

Response 1. We thank the reviewer for this comment. The manuscript has been carefully revised to improve readability by simplifying long and complex sentences, correcting minor grammatical inconsistencies, and eliminating redundancies.

Comment 2. Maintain consistent use of units, abbreviations, and spectral terminology throughout the manuscript.

Response 2. We thank the reviewer for this helpful comment. The manuscript has been carefully revised to ensure consistent use of units, abbreviations, and spectral terminology throughout the text.

Comment 3. Consider incorporating a few more recent (last 2–3 years) references on LED optimization in microgreens to strengthen the literature positioning.

Response 3. We thank the reviewer for this suggestion. In response, recent references on LED optimization in microgreens have been incorporated into the manuscript, including one study from 2025 and three studies from 2026. The relevant references are listed below.

Da Young, L. E. E., Kim, M. J., In-Lee, C. H. O. I., Yongduk, K. I. M., Jidong, K. I. M., & Ho-Min, K. A. N. G. (2025). Effect of LED light quality on the growth and photomorphogenesis of basil in closed-type plant factory. Notulae Botanicae Horti Agrobotanici Cluj-Napoca, 53(2), 14409-14409.

Bafrani, M. V. A., Mehrjerdi, M. Z., Aliniaeifard, S., & Seif, M. (2026). Optimizing LED light in controlled environments to enhance yield and nutritional quality of basil microgreens. South African Journal of Botany, 190, 262-275.

Sobhanan, A., Meena, R. K., Dhaka, A. S., Mishra, G. P., & Nishad, J. (2026). Optimization of LED-Based Phototreatments for Enhancing Antioxidant Potential and Marketable Traits in Microgreens. Applied Food Research, 101918. SOBHANAN

Borrelli, A., Zamani, F., Logegaray, V.R. et a

Attachment Submitted filename: Response to reviewers April 7, 2026.docx

# Editor report: Decision Letter 2

**DOI:** 10.1371/journal.pone.0352317.r005

**Authors:** Partha Kalita, Branislav Šiler, Branislav Šiler

26 Apr 2026

<div>PONE-D-25-35263R2

Effects of LED Spectral Compositions on Yield, Growth, and Nutritional Quality of Basil Microgreens in Indoor Vertical Farming

PLOS One

Dear Dr. Dasgan,

Thank you for submitting your manuscript to PLOS ONE. After careful consideration, we feel that it has merit but does not fully meet PLOS ONE’s publication criteria as it currently stands. Therefore, we invite you to submit a revised version of the manuscript that addresses the points raised during the review process.

Please submit your revised manuscript by Jun 10 2026 11:59PM. If you will need more time than this to complete your revisions, please reply to this message or contact the journal office at plosone@plos.org. When you're ready to submit your revision, log on to https://www.editorialmanager.com/pone/ and select the 'Submissions Needing Revision' folder to locate your manuscript file.

Please include the following items when submitting your revised manuscript:

- A letter that responds to each point raised by the academic editor and reviewer(s). You should upload this letter as a separate file labeled 'Response to Reviewers'.
- A marked-up copy of your manuscript that highlights changes made to the original version. You should upload this as a separate file labeled 'Revised Manuscript with Track Changes'.
- An unmarked version of your revised paper without tracked changes. You should upload this as a separate file labeled 'Manuscript'.

If you would like to make changes to your financial disclosure, please include your updated statement in your cover letter. Guidelines for resubmitting your figure files are available below the reviewer comments at the end of this letter.

If applicable, we recommend that you deposit your laboratory protocols in protocols.io to enhance the reproducibility of your results. Protocols.io assigns your protocol its own identifier (DOI) so that it can be cited independently in the future. For instructions see: https://journals.plos.org/plosone/s/submission-guidelines#loc-laboratory-protocols. Additionally, PLOS ONE offers an option for publishing peer-reviewed Lab Protocol articles, which describe protocols hosted on protocols.io. Read more information on sharing protocols at https://plos.org/protocols?utm_medium=editorial-email&utm_source=authorletters&utm_campaign=protocols.

As the corresponding author, your ORCID iD is verified in the submission system and will appear in the published article. PLOS supports the use of ORCID, and we encourage all coauthors to register for an ORCID iD and use it as well. Please encourage your coauthors to verify their ORCID iD within the submission system before final acceptance, as unverified ORCID iDs will not appear in the published article. *Only*  the individual author can complete the verification step; PLOS staff *cannot*  verify ORCID iDs on behalf of authors.

We look forward to receiving your revised manuscript.

Kind regards,

Branislav T. Šiler, Ph.D.

Academic Editor

PLOS One

Journal Requirements:

If the reviewer comments include a recommendation to cite specific previously published works, please review and evaluate these publications to determine whether they are relevant and should be cited. There is no requirement to cite these works unless the editor has indicated otherwise.

Please review your reference list to ensure that it is complete and correct. If you have cited papers that have been retracted, please include the rationale for doing so in the manuscript text, or remove these references and replace them with relevant current references. Any changes to the reference list should be mentioned in the rebuttal letter that accompanies your revised manuscript. If you need to cite a retracted article, indicate the article’s retracted status in the References list and also include a citation and full reference for the retraction notice.

Additional Editor Comments:

Both reviewers have refrained from further comments, but I find most of their concerns properly addressed in the revised manuscript. One problematic point is Figure 5, where the three LED sources were tested against the color (L*, a*, and b*). It seems that statistical testing has failed here, since large standard errors may not guarantee statistical differences. Please re-check your data.

[Note: HTML markup is below. Please do not edit.]

[NOTE: If reviewer comments were submitted as an attachment file, they will be attached to this email and accessible via the submission site. Please log into your account, locate the manuscript record, and check for the action link "View Attachments". If this link does not appear, there are no attachment files.]

To ensure your figures meet our technical requirements, please review our figure guidelines: https://journals.plos.org/plosone/s/figures

You may also use PLOS’s free figure tool, NAAS, to help you prepare publication quality figures: https://journals.plos.org/plosone/s/figures#loc-tools-for-figure-preparation.

NAAS will assess whether your figures meet our technical requirements by comparing each figure against our figure specifications.

# Author comment: Author response to Decision Letter 3

**DOI:** 10.1371/journal.pone.0352317.r006

22 May 2026

May 15, 2026

Dear Editor Dr. Šiler,

Thank you very much for your valuable comment and careful evaluation of our manuscript. We sincerely appreciate your observation regarding the visual appearance of the error bars in Figure 5. Following your recommendation, we carefully re-examined both the statistical outputs and the graphical presentation of the data. The issue you highlighted has been addressed in detail below, and the figure has been revised accordingly.

In Figure 5, the error bars in the Color L* and Color b* graphs appeared visually large. Upon re-examining the data, we confirmed that the standard error values were not actually high and that the replicates were relatively close to each other. However, we noticed that the visual appearance of the error bars was mainly caused by the scaling of the y-axis during graph preparation.

In the previous version (Old Version of Figure 5), the y-axis values were displayed at very narrow intervals. For example, in the Color L* graph, the scale was arranged as 86, 86.5, 87, 87.5, etc., while in the Color b* graph it was arranged as 0, 0.5, 1.0, 1.5, 2.0, etc. Such narrow intervals visually exaggerated the error bars.

Therefore, the graphs have now been revised (New Version Figure 5). In the updated Color L* graph, the y-axis values are presented at intervals of 5 units (50, 55, 60, 65, 70, 75, etc.). Similarly, in the Color a* and Color b* graphs, the y-axis values are now presented at intervals of 1 unit (0, 1, 2, 3, 4, 5, 6, etc.). In this revised form, the standard error bars no longer appear artificially inflated as in the previous version.

Since there were no changes in the original data or statistical outputs related to the color parameters (L*, a*, and b*), no revisions were required in the Results or Discussion sections of the manuscript. Accordingly, only Figure 5 was revised in the current submission.

We respectfully submit these revisions for your consideration and kindly hope that the manuscript can now be accepted for publication in its final revised form.

Sincerely,

Prof. Dr. H. Yildiz Dasgan

Corresponding author

Attachment Submitted filename: Response to editor comment May 22, 2026.docx

# Editor report: Decision Letter 3

**DOI:** 10.1371/journal.pone.0352317.r007

**Authors:** Partha Kalita, Branislav Šiler, Branislav Šiler, Branislav Šiler

26 May 2026

PONE-D-25-35263R3

Effects of LED Spectral Compositions on Yield, Growth, and Nutritional Quality of Basil Microgreens in Indoor Vertical Farming

PLOS One

Dear Dr. Dasgan,

Thank you for submitting your manuscript to PLOS ONE. After careful consideration, we feel that it has merit but does not fully meet PLOS ONE’s publication criteria as it currently stands. Therefore, we invite you to submit a revised version of the manuscript that addresses the points raised during the review process.

Please submit your revised manuscript by Jul 10 2026 11:59PM. If you will need more time than this to complete your revisions, please reply to this message or contact the journal office at plosone@plos.org. When you're ready to submit your revision, log on to https://www.editorialmanager.com/pone/ and select the 'Submissions Needing Revision' folder to locate your manuscript file.

Please include the following items when submitting your revised manuscript:

- A letter that responds to each point raised by the academic editor and reviewer(s). You should upload this letter as a separate file labeled 'Response to Reviewers'.
- A marked-up copy of your manuscript that highlights changes made to the original version. You should upload this as a separate file labeled 'Revised Manuscript with Track Changes'.
- An unmarked version of your revised paper without tracked changes. You should upload this as a separate file labeled 'Manuscript'.

If you would like to make changes to your financial disclosure, please include your updated statement in your cover letter. Guidelines for resubmitting your figure files are available below the reviewer comments at the end of this letter.

If applicable, we recommend that you deposit your laboratory protocols in protocols.io to enhance the reproducibility of your results. Protocols.io assigns your protocol its own identifier (DOI) so that it can be cited independently in the future. For instructions see: https://journals.plos.org/plosone/s/submission-guidelines#loc-laboratory-protocols. Additionally, PLOS ONE offers an option for publishing peer-reviewed Lab Protocol articles, which describe protocols hosted on protocols.io. Read more information on sharing protocols at https://plos.org/protocols?utm_medium=editorial-email&utm_source=authorletters&utm_campaign=protocols.

As the corresponding author, your ORCID iD is verified in the submission system and will appear in the published article. PLOS supports the use of ORCID, and we encourage all coauthors to register for an ORCID iD and use it as well. Please encourage your coauthors to verify their ORCID iD within the submission system before final acceptance, as unverified ORCID iDs will not appear in the published article. *Only*  the individual author can complete the verification step; PLOS staff *cannot*  verify ORCID iDs on behalf of authors.

We look forward to receiving your revised manuscript.

Kind regards,

Branislav T. Šiler, Ph.D.

Academic Editor

PLOS One

Journal Requirements:

If the reviewer comments include a recommendation to cite specific previously published works, please review and evaluate these publications to determine whether they are relevant and should be cited. There is no requirement to cite these works unless the editor has indicated otherwise.

Please review your reference list to ensure that it is complete and correct. If you have cited papers that have been retracted, please include the rationale for doing so in the manuscript text, or remove these references and replace them with relevant current references. Any changes to the reference list should be mentioned in the rebuttal letter that accompanies your revised manuscript. If you need to cite a retracted article, indicate the article’s retracted status in the References list and also include a citation and full reference for the retraction notice.

Additional Editor Comments:

New Figure 5 is of very low resolution. Please follow the PLOS ONE Figure preparation guidelines at: https://journals.plos.org/plosone/s/figures. Not all lines are visible in Figure 6B.

[Note: HTML markup is below. Please do not edit.]

[NOTE: If reviewer comments were submitted as an attachment file, they will be attached to this email and accessible via the submission site. Please log into your account, locate the manuscript record, and check for the action link "View Attachments". If this link does not appear, there are no attachment files.]

To ensure your figures meet our technical requirements, please review our figure guidelines: https://journals.plos.org/plosone/s/figures

You may also use PLOS’s free figure tool, NAAS, to help you prepare publication quality figures: https://journals.plos.org/plosone/s/figures#loc-tools-for-figure-preparation.

NAAS will assess whether your figures meet our technical requirements by comparing each figure against our figure specifications.

# Author comment: Author response to Decision Letter 4

**DOI:** 10.1371/journal.pone.0352317.r008

8 Jun 2026

Dear Editor,

Thank you for your comments. We have addressed all requested revisions. The resolution of Figure 5 has been improved, and the unclear lines in Figure 6 have been corrected. The revised figures have been incorporated into the manuscript, and the updated manuscript file has been uploaded to the system.

A detailed point-by-point response to the editorial comments has also been provided and uploaded as a separate file.

Thank you for your consideration.

Sincerely,

Prof. Dr. H. Yıldız Daşgan

Corresponding Author

Attachment Submitted filename: RESPONSE TO EDITORIAL COMMENTS.docx

# Editor report: Decision Letter 4

**DOI:** 10.1371/journal.pone.0352317.r009

**Authors:** Partha Kalita, Branislav Šiler, Branislav Šiler, Branislav Šiler, Branislav Šiler

9 Jun 2026

Effects of LED Spectral Compositions on Yield, Growth, and Nutritional Quality of Basil Microgreens in Indoor Vertical Farming

PONE-D-25-35263R4

Dear Dr. Dasgan,

We’re pleased to inform you that your manuscript has been judged scientifically suitable for publication and will be formally accepted for publication once it meets all outstanding technical requirements.

Within one week, you’ll receive an e-mail detailing the required amendments. When these have been addressed, you’ll receive a formal acceptance letter and your manuscript will be scheduled for publication.

An invoice will be generated when your article is formally accepted. Please note, if your institution has a publishing partnership with PLOS and your article meets the relevant criteria, all or part of your publication costs will be covered. Please make sure your user information is up-to-date by logging into Editorial Manager at Editorial Manager® and clicking the ‘Update My Information' link at the top of the page. For questions related to billing, please contact billing support.

If your institution or institutions have a press office, please notify them about your upcoming paper to help maximize its impact. If they’ll be preparing press materials, please inform our press team as soon as possible -- no later than 48 hours after receiving the formal acceptance. Your manuscript will remain under strict press embargo until 2 pm Eastern Time on the date of publication. For more information, please contact onepress@plos.org.

Kind regards,

Branislav T. Šiler, Ph.D.

Academic Editor

PLOS One

Additional Editor Comments (optional):

Reviewers' comments:

# Editor report: Acceptance letter

**DOI:** 10.1371/journal.pone.0352317.r010

**Authors:** Partha Kalita, Branislav Šiler, Branislav Šiler, Branislav Šiler, Branislav Šiler

PONE-D-25-35263R4

PLOS One

Dear Dr. Gruda,

I'm pleased to inform you that your manuscript has been deemed suitable for publication in PLOS One. Congratulations! Your manuscript is now being handed over to our production team.

At this stage, our production department will prepare your paper for publication. This includes ensuring the following:

* All references, tables, and figures are properly cited

* All relevant supporting information is included in the manuscript submission,

* There are no issues that prevent the paper from being properly typeset

You will receive further instructions from the production team, including instructions on how to review your proof when it is ready. Please keep in mind that we are working through a large volume of accepted articles, so please give us a few days to review your paper and let you know the next and final steps.

Lastly, if your institution or institutions have a press office, please let them know about your upcoming paper now to help maximize its impact. If they'll be preparing press materials, please inform our press team within the next 48 hours. Your manuscript will remain under strict press embargo until 2 pm Eastern Time on the date of publication. For more information, please contact onepress@plos.org.

You will receive an invoice from PLOS for your publication fee after your manuscript has reached the completed accept phase. If you receive an email requesting payment before acceptance or for any other service, this may be a phishing scheme. Learn how to identify phishing emails and protect your accounts at https://explore.plos.org/phishing.

If we can help with anything else, please email us at customercare@plos.org.

Thank you for submitting your work to PLOS ONE and supporting open access.

Kind regards,

PLOS ONE Editorial Office Staff

on behalf of

Dr. Branislav T. Šiler

Academic Editor

PLOS One
